# Therapeutic Potential of Gasdermin D‐Mediated Myocardial Pyroptosis in Ischaemic Heart Disease: Expanding the Paradigm From Bench to Clinical Insights

**DOI:** 10.1111/jcmm.70357

**Published:** 2025-02-10

**Authors:** Chanon Piamsiri, Chayodom Maneechote, Siriporn C. Chattipakorn, Nipon Chattipakorn

**Affiliations:** ^1^ Cardiac Electrophysiology Research and Training Center, Faculty of Medicine Chiang Mai University Chiang Mai Thailand; ^2^ Cardiac Electrophysiology Unit, Department of Physiology, Faculty of Medicine Chiang Mai University Chiang Mai Thailand; ^3^ Center of Excellence in Cardiac Electrophysiology Research Chiang Mai University Chiang Mai Thailand; ^4^ Department of Oral Biology and Diagnostic Sciences, Faculty of Dentistry Chiang Mai University Chiang Mai Thailand

**Keywords:** heart failure, ischaemic heart disease, myocardial infarction, programmed cell death, pyroptosis

## Abstract

Ischaemic heart disease (IHD) remains a leading cause of global morbidity and mortality. One significant contributor to the pathology of IHD is the excessive release of inflammatory mediators during the disease progression. Pyroptosis is a form of programmed cell death (PCD) triggered by the activation of inflammasomes and caspase 1. The activation of inflammatory caspase 1 proteolytically cleaves gasdermin D (GSDMD) to the activated form amino acid terminus (GSDMD‐NT), leading to disruption of the plasma membrane. This cascade of events is considered the canonical pathway of pyroptosis. IHD also caused oxidative stress, thereby triggering noncanonical pyroptosis via the activation of caspases 4/5/11. Previous studies have provided compelling evidence of the close relationship between pyroptosis and the aetiology of IHD (e.g., acute myocardial infarction, myocardial ischaemia and reperfusion injury and chronic myocardial infarction), as well as the association of pyroptosis with unfavourable clinical outcomes. Several interventions aimed at targeting pyroptosis have demonstrated promising therapeutic benefits against IHD‐related pathologies. This review provides mechanistic insights into the roles of pyroptosis in IHD from in vitro, in vivo and clinical perspectives. In‐depth understanding into this area could also pave the way for the future development of novel therapeutic strategies targeting pyroptosis in IHD.

**Abbreviations**
ASC: apoptosis‐associated speck‐like protein containing a CARD
DAMPs: damage‐associated molecular patterns
ECM: extracellular matrix
GSDMD: gasdermin D
GSDMD‐NT: *N*‐terminal domain of gasdermin D
GSDME: gasdermin E
HMGB1: high mobility group box 1
IHD: ischaemic heart disease
IKKα/β: IKK alpha kinase α/β
IL‐18: interleukin 18
IL‐1β: interleukin 1β
mtDNA: mitochondrial DNA
mtROS: mitochondrial ROS
Myd88: myeloid differentiation factor 88
NAC: *N*‐acetylcysteine
NF‐κB: nuclear factor kappa B
NLRP3: nucleotide‐binding domain, leucine‐rich‐containing family, pyrin domain‐containing‐3
PRRs: pathogen recognition receptors
TAK1: transforming growth factor‐β activated kinase 1
TLR4: toll‐like receptor 4
TNFR: tumour necrosis factor receptor‐associated factor
TNF‐α: tumour necrosis factor alpha

## 1 Introduction

Ischaemic heart disease (IHD), synonymously called coronary heart disease (CHD), is recognised as the foremost cause of morbidity and the condition predominantly underlying the incidence of sudden death worldwide [1]. IHD is predominantly caused by the blockage of the coronary vessels, which is most often attributed to the deposition of atherosclerotic plaque, leading to consequential death of the myocardium [2, 3]. IHD can manifest itself in both acute and chronic forms [4]. Acute ischaemic heart disease occurs as a result of the abrupt obstruction of coronary blood flow, typically caused by coronary thrombosis or the rupture of an atherosclerotic plaque [5]. This acute blockage subsequently leads to the development of acute myocardial infarction (AMI), or a heart attack [6]. In contrast, chronic ischaemic heart disease develops gradually over time due to the progressive disruption or blockage of coronary perfusion [2, 3, 5]. This condition is often referred to as stable ischaemic heart disease, chronic coronary artery disease, post‐MI or chronic myocardial infarction (CMI) [2, 3, 5]. Fortunately, cardiac revascularisation, considered the mandatory treatment of choice for patients with IHD, substantially reduces in‐hospital mortality among IHD patients [7, 8]. However, the restoration of blood flow to the obstructed coronary vessels is also known to paradoxically cause further damage to the myocardium, attributable to oxidative stress, inflammation and a surge in cytokine activity. This phenomenon is referred to as cardiac ischaemia and reperfusion (I/R) injury [9]. The estimation for the adult human cardiomyocyte turnover rate is about 0.5%–2% per year, which is relatively insufficient to replace the substantial loss of functional cells following ischaemic injury [10, 11]. Irreversible damage to the myocardium usually leads to myocardial death, pathological remodelling, contractile dysfunction and the development of heart failure [12, 13].

It has been shown that the initiation of pyroptosis is associated with the development of several IHDs and is considered as one of the most potent programmed cell death (PCD) mechanisms among the various kinds of IHD [14, 15, 16, 17, 18, 19]. Pyroptosis, initially identified by Zychlinsky, Prevost, and Sansonetti [20], manifests as a cellular response to lipopolysaccharides (LPS) produced by gram‐negative bacteria, leading to lytic cell death in macrophages. Pyroptosis was previously known as a proinflammatory, programmed type of cell death characterised by the activation of the inflammasomes along with the proteolytic cleavage of gasdermin proteins [15, 18, 21]. During the past decade, an increasing number of studies have demonstrated that under certain IHD conditions (i.e., AMI, cardiac I/R and post‐MI scenarios), augmented activation of pyroptosis activation is responsible for worsening clinical and functional outcomes [15, 22]. On the contrary, the inhibition of pyroptosis showed potential therapeutic efficacy against IHDs in the preclinical settings [14, 23, 24]. This comprehensive review provides insights into the molecular mechanisms of pyroptosis‐mediated myocardial death in IHD, and their potential as targets for future therapeutic strategies for improving clinical outcomes in IHD patients.

## 2 Pyroptosis in IHD

Pyroptosis is a form of programmed cell death intimately controlled by proinflammatory pathways, and vice versa [21, 25]. It has been reported that IHD has the potential to develop cardiac pyroptosis through both canonical and noncanonical pathways. In the case of canonical pyroptosis, several endogenous DAMPs (e.g., tumour necrosis factor‐α or TNF‐α, high mobility group box 1 or HMGB1 and mitochondrial DNA or mtDNA) are discharged into extracellular space and induce inflammasome priming through the activation PRRs such as toll‐like receptor 4 (TLR4) [15]. The priming of inflammasome‐mediated canonical pyroptosis predominantly occurs through TLR4/Myd88/NF‐κB/p65 signalling cascades [22, 26]. Moreover, oxidative stress and oxidised mtDNA may directly promote the activation of inflammasomes, leading to the activation of canonical pyroptosis [27]. One of the most common characteristics of IHD is the overwhelming level of inflammation and excessive release of DAMPs [28].

In both the ischaemic episode and the subsequent reperfusion periods, several endogenous DAMPs (e.g., TNF‐α, HMGB1 or mtDNA) are generated by the neighbouring necrotic cells and discharged into the extracellular space [15, 28, 29, 30]. Under physiological conditions, these DAMPs are typically absent in the normal interstitium [31]. However, in cases of IHDs, the presence of these endogenous danger signals has the potential to initiate inflammasome priming by binding and activating their respective PRRs, such as the interaction between TNF‐α and HMGB1 with TLR4 [22, 26]. Priming of TLR4 leads to the subsequent activation and translocation of nuclear factor kappa B (NF‐κB) [22, 26]. The nuclear translocation of NF‐κB results in transcriptional upregulation of the expression of procaspase 1 and also canonical inflammasome components, including the nucleotide‐binding domain, the leucine‐rich‐containing family, pyrin domain‐containing‐3 (NLRP3) and the adaptor molecule apoptosis‐associated speck‐like protein containing CARD (ASC) [15, 22, 26]. The activation of NLRP3, in turn, recruits the adapter protein ASC, forming a NLRP3 inflammasome [15, 22, 26]. Furthermore, the assembly of the NLRP3 inflammasome causes the cleavage of procaspase 1 into its activated form, cleaved caspase 1 and facilitates the production of IL‐1β and IL‐18 [15, 22].

**FIGURE 1:**
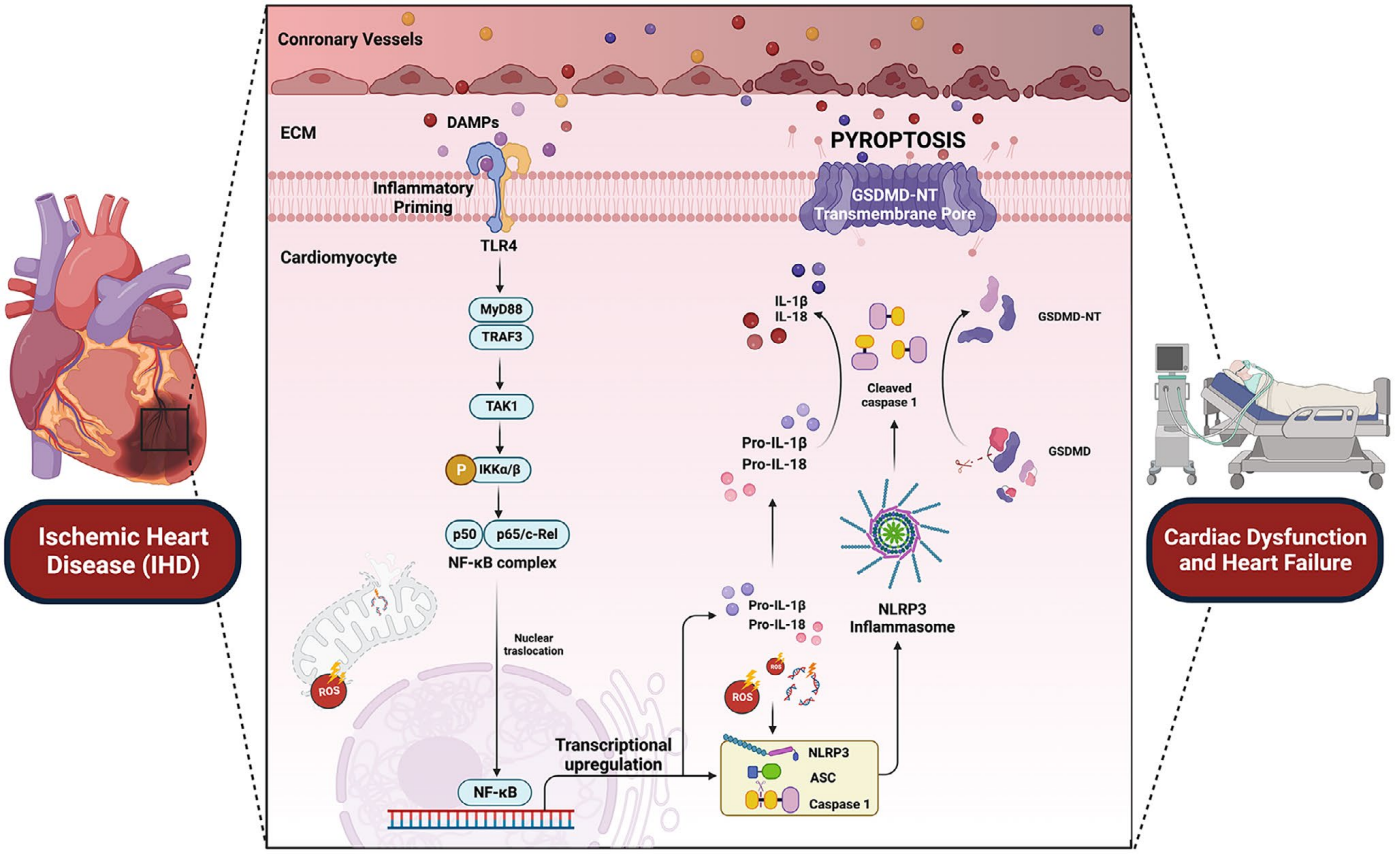
Pyroptosis signalling cascade in IHD involves a series of events. During ischaemic stress and the reperfusion period several DAMPs such as TNF‐α, HMGB1 and mtDNA bind to TLR4 on the plasma membrane. This interaction primes TLR4, leading to subsequent activation of the TLR4/MyD88/TAK1/NF‐κB signalling pathway. Activated NF‐κB translocases into the nucleus, resulting in the transcriptional upregulation of pro‐caspase 1, NLRP3, ASC, pro‐IL‐1β and pro‐IL‐18. NLRP3 recruits the adapter protein ASC, forming the NLRP3 inflammasome. mtROS and oxidised mtDNA can also directly interact with NLRP3, facilitating the assembly of the inflammasome and the subsequent activation of caspase 1. Assembly of the NLRP3 inflammasome causes the cleavage of procaspase 1 into its active form, cleaved caspase 1. Once activated, caspase 1 further cleaves pro‐IL‐1β into IL‐1β and pro‐IL‐18 into IL‐18. Cleaved caspase 1 also proteolytically cleaves GSDMD into GSDMD‐NT. GSDMD‐NT undergoes oligomerisation and translocates to the inner leaflet of the plasma membrane, leading to the formation of GSDMD‐NT transmembrane pores. These pores allow for the influx of extracellular substances (e.g., extracellular Ca^2+^ and H_2_O) and the efflux of intracellular components (e.g., IL‐1β, IL‐18 and K^+^), ultimately culminating in pyroptosis execution. Consequently, IHD‐mediated myocardial pyroptosis results in myocardial cell death, inflammation, cardiac injury, adverse cardiac remodelling, contractile dysfunction and the development of heart failure. ASC, apoptosis‐associated speck‐like protein containing a CARD; DAMPs, damage‐associated molecular patterns; ECM, extracellular matrix; GSDMD, gasdermin D; GSDMD‐NT, *N*‐terminal domain of gasdermin D; IHD, ischaemic heart disease; IKKα/β, IKKalpha kinase α/β; IL‐18, interleukin‐18; IL‐1β, interleukin 1β; Myd88, myeloid differentiation factor 88; NF‐κB, nuclear factor kappa B; NLRP3, nucleotide‐binding domain, leucine‐rich‐containing family, pyrin domain‐containing‐3; TAK1, transforming growth factor‐β activated kinase 1; TRAF3, tumour necrosis factor receptor‐associated factor 3.

Under physiological conditions, the full‐length gasdermin D (GSDMD) is autoinhibited and acts as a nonfunctional protein [32, 33]. Conversely, under pathological conditions, including IHD (i.e., AMI, cardiac I/R and post‐MI scenarios), the overwhelming activation of inflammatory caspase 1 proteolytically cleaves GSDMD to generate the carboxylic terminus (*C*‐terminal domain or GSDMD‐CT) and amino acid terminus (*N*‐terminal domain or GSDMD‐NT) [15, 22]. Crucially, GSDMD‐NT acts as a functionally active component [15, 22]. GSDMD‐NT preferentially translocases and undergoes oligomerisation, then targets phosphatidylinositol phosphates and phosphatidylserine in the inner leaflet of the plasma membrane, resulting in the formation of transmembrane pores [15, 21, 22, 28, 29, 30]. It has been shown that the binding of GSDMD‐CT with GSDMD‐NT inhibits their activation [34]. The GSDMD‐NT transmembrane pores range from 10 to 20 nm, which not only allows the efflux of intracellular components (e.g., IL‐1β, IL‐18 and K^+^) but also facilitates the influx of extracellular materials (e.g., extracellular Ca^2+^ and H_2_O) [15, 22]. Consequently, these insults increase intracellular osmotic pressure and cellular swelling, ultimately resulting in osmotic lysis and the release of intracellular contents [35]. This process is considered the execution event of pyroptosis, which induces cell death [15, 22]. The roles of canonical pyroptosis in IHD are summarised in Figure 1. In IHD, pyroptosis has been shown to amplify the inflammatory response, worsen myocardial injury and infarct progression and accelerate the development of heart failure [36, 37]. Pyroptosis plays a central role in sterile inflammation by triggering the release of endogenous DAMPs, such as TNF‐α, HMGB1 and mtDNA, from neighbouring necrotic cells [25, 38, 39]. The activation of GSDMD pores further amplifies inflammation, worsening cardiomyocyte death and impairing systolic function [39]. In IHD, including AMI, cardiac I/R and post‐MI conditions, pyroptosis contributes to myocardial cell death and contractile dysfunction, ultimately leading to heart failure with reduced ejection fraction (HFrEF). Pyroptosis directly leads to the death of the functional cardiomyocytes, which affecting both systolic and diastolic functions of the myocardium and promoting cardiac contractile dysfunction in HFrEF [36, 40]. Previously, Prescimone et al. [41] reported the evidence of a distinct localised apoptotic transcriptional profile in a swine model of heart failure with preserved ejection fraction (HFpEF). However, in HFpEF, there is limited evidence reported that pyroptosis activation also contributes to the progression of heart failure, despite the absence of systolic dysfunction. A previous study by Xia et al. [42] provided evidence of pyroptosis in myocardial tissue and epicardial adipose tissue in HFpEF mice, induced by a two‐hit protocol (western diet and Nω‐nitro‐L‐arginine methyl ester (L‐NAME) administration). They found that the activation of NLRP3‐mediated pyroptosis of epicardial adipose tissue caused myocardial inflammation in HFpEF mice [42]. Additionally, Cheng et al. [43] has reported the cardioprotective effects of inflammasome inhibitor (MCC950) in a mouse model of HFpEF induced by two‐hit protocols. MCC950 effectively inhibited NLRP3‐mediated pyroptosis thus reduced cardiac inflammation and remodelling in HFpEF mice [43]. In summary, pyroptosis plays a critical role in the pathophysiology of both HFrEF and HFpEF, by exacerbating myocardial injury, inflammation, and contractile dysfunction. However, the contribution of pyroptosis in HFpEF remains incompletely understood, underscoring the need for further studies to explore this aspect.

## 3 Evidence of Canonical Pyroptosis in IHD: Reports Across In Vitro Studies

In in vitro studies, cardiomyocytes were cultured under various stress conditions, including hypoxic and hypoxia reoxygenation (H/R) models. In both hypoxic and H/R conditions, pyroptotic death could be induced in cultured cardiomyocytes by the upregulation of the TLR4/Myd88/NF‐κB/p65 signalling pathway, resulting in a significant increase in key pyroptosis proteins including NLRP3, ASC, cleaved caspase 1 and GSDMD‐NT, leading to cardiomyocyte injury and decreased cell viability [44, 45, 46]. Likewise, IL‐1β and IL‐18 have also been found to be increased in cultured cardiomyocytes (e.g., H9c2 cells), neonatal mouse ventricular cardiomyocytes or NMVMs and neonatal rat ventricular cardiomyocytes (NRVMs) under hypoxic and H/R insults [44, 45, 46]. Since GSDMD serves as the pyroptosis executioner molecule, genetic ablation of GSDMD effectively mitigates oxidative stress‐induced pyroptosis in cultured isolated cardiomyocytes [17, 45]. In contrast, GSDMD overexpression exacerbated cell injury and further augmented pyroptosis in cardiomyocytes under H/R conditions [45]. Hypoxia and H/R‐induced cardiomyocyte pyroptosis has also been found to be related to augmented oxidative stress and oxidative stress enzymes (i.e., MDA and NOX4) and also a decrease in antioxidative enzyme levels (i.e., GSH and SOD) [44, 45, 46, 47, 48]. Although GSDMD is recognised as damaging to both the plasma membrane and mitochondria during pyroptosis, recent studies have also revealed that the impairment of mitochondrial function and alterations in mitochondrial‐related proteins also contribute to pyroptosis [49, 50].

An increasing body of experimental evidence indicates that the advancement of IHD correlates with a deterioration in cardiac mitochondrial function through metabolic shifts, intracellular acidosis, Ca^2+^ overload and oxidative stress [27, 51, 52, 53, 54]. In an in vitro study using the isolated adult rat cardiomyocytes, H/R insults were found to enhance both maximal intracellular and maximal mitochondrial Ca^2+^ levels, augment mitochondrial ROS (mtROS) and alter mitochondrial membrane potential (ΔΨm) [55]. Subsequently, the impairment of mitochondrial function and mitochondrial damage occurred as a result of cardiomyocyte pyroptosis through the NLRP3/GSDMD signalling cascade. Wang et al. [56] reported that the expression of proprotein convertase subtilisin/kexin type 9 (PCSK9) was escalated in hypoxic cultured NMVMs, leading to mtDNA damage, mtROS overproduction and pyroptotic death. It has been shown that oxidative stress and oxidised mtDNA could directly promote the activation of inflammasomes, leading to the activation of caspase 1 and pyroptotic execution [15]. In contrast, PCSK9 knockout could attenuate mitochondrial DNA damage and excessive mtROS production, mitigating the activation of pyroptosis in cardiomyocytes under hypoxic conditions [56].

Mitofilin, an important protein for the maintenance of mitochondrial cristae structure and remodelling, was found to be decreased in H/R cardiomyocytes [46]. Knockdown of mitofilin exacerbated H/R‐induced cardiomyocyte pyroptosis, while its overexpression protected against H/R‐induced cardiomyocyte pyroptosis through the phosphatidylinositol 3′‐kinase/protein kinase B (PI3K/AKT)‐dependent pathway [46]. In addition, H/R also mediated the overactivation of inositol trisphosphate receptors (IP3Rs) and the reduction in their inhibitory protein ERP44, leading to Ca^2+^ overload, thus triggering pyroptosis [55]. The presence of intracellular Ca^2+^ overload is widely recognised as a prerequisite for NLRP3 activation and the subsequent induction of pyroptosis [57, 58]. Furthermore, either knockout of IP3R1 or overexpression of ERP44 could mitigate Ca^2+^ overload, inflammation and pyroptosis in cardiomyocytes following H/R insults [55]. Moreover, in the presence of prolonged hypoxic stress, heightened cardiac mtDNA oxidisation and increased mtROS also promoted GSDMD‐mediated cardiomyocyte pyroptosis [56]. The interplay of these molecular cascades highlights the multifaceted mechanisms by which dysregulation of cardiac mitochondrial function influences pyroptotic processes in cardiomyocytes subjected to ischaemic mimicking insults. In addition to Ca^2+^ and oxidative stress, retinol binding protein 4 (RBP4), a proinflammatory adipokine, was discovered to be upregulated in H/R cardiomyocytes, significantly exacerbating canonical pyroptosis by interacting with NLRP3 [48]. Knockdown of RPB4 also attenuated cardiomyocyte pyroptosis through inhibition of the NLRP3 signalling pathway in hypoxic cardiomyocytes [48].

Recently, several studies have been conducted to investigate the involvement of noncoding ribonucleic acids (RNAs), including microRNA (miR) and long noncoding RNA (LncRNA), in the context of both IHD and pyroptosis. It has been shown that knockdown of LncRNA PVT1 and LncRNA MEG3 rescued H/R‐induced pyroptosis in hypoxic cardiomyocytes [59, 60]. Previous studies reported that LncRNA FAF and LncRNA H19, which are known as the regulators of prosurvival signalling, were found to be downregulation during ischaemia and hypoxia [39, 40, 42, 63, 64, 65]. Additionally, it has been shown that the downregulation of LncRNA FAF led to cardiomyocyte apoptosis and pyroptosis [39, 43, 66]. Interestingly, overexpression of LncRNA H19 and LncRNA FAF conveyed cytoprotective effects against NLRP3/Caspase 1/GSDMD‐NT‐mediated pyroptosis in hypoxic cardiomyocytes [39, 44].

Interestingly, overexpression of LncRNA H19 and LncRNA FAF conveyed cytoprotective effects against hypoxia‐induced pyroptosis in cultured cardiomyocytes [61, 67]. These initial findings demonstrated the roles of LncRNA in IHD‐related pathogenesis. However, it is important to recognise the roles of noncoding RNAs and their downstream signalling to enhance our understanding of IHD‐mediated pyroptosis pathogenesis. Collectively, hypoxia and H/R insults induce cardiomyocyte oxidative stress, dysregulation of mitochondrial and mitochondrial‐related proteins, Ca^2+^ overload and noncoding RNA alteration. Subsequently, this cascade triggers canonical pyroptosis and inflammation via the NLRP3/caspase 1/GSDMD‐NT signalling pathway. A summary of canonical pyroptosis in the in vitro models of IHDs is shown in Table 1.

**TABLE 1:** Pyroptosis in IHD models: reports across the in vitro studies.

Study models	Methods	Major findings				Interpretation	Reference
		Cell viability/functional outcome	Pyroptosis	Inflammation	Other biochemical markers		
NMVMs cells	H/R (H: 0.5 h, R: 24 h)	↑ LDH cytotoxicity	↑ Caspase1 ↑ Cleaved caspase1 ↑ GSDMD ↑ GSDMD‐NT ↔ GSDME	↑ IL‐18	↑ PI‐positive nuclei ↓ ATP	H/R‐induced cardiomyocyte pyroptosis through cleaved caspase 11/GSDMD—but not GSDME activation.	[17]
NMVMs cells	GSDMD knockout + H_2_O_2_ 200 μM for 2 h (compared with wildtype)	↓ LDH cytotoxicity	↓ GSDMD ↓ GSDMD‐NT	N/A	↓ PI‐positive nuclei ↑ ATP	Oxidative stress‐induced cardiomyocyte pyroptosis thought GSDMD activation.	[17]
NRVMs cells	H/R (H: 1 h, R: 2 h)	↓ Cell viability ↑ LDH cytotoxicity	↑ NLRP3 ↑ ASC ↑ Cleaved caspase 1 ↔ GSDMD ↑ GSDMD‐NT	↑ IL‐1β	↑ TLR4 ↑ MyD88 ↑ p‐p65/p65 ↑ p‐iκBα/iκBα ↑ MDA ↑ DHE ↓ SOD	H/R‐mediated ROS overproduction and induced cardiomyocyte pyroptosis via Tlr4/MyD88/NF‐κB/NLRP3 inflammasome pathway.	[68]
HCMECs cells	H/R (H: 2 h, R: 2 h)	↓ Cell viability ↑ LDH cytotoxicity	↑ GSDMD‐NT ↑ Caspase 4	N/A	↑ Beclin‐1, LC3 II/I, p62	H/R aggravated autophagy flux impairment and enhanced cardiac microvascular endothelial cells pyroptosis. sRAGE mitigated pyroptosis in cultured cardiac microvascular endothelial cells following H/R insults though the regulation of autophagy flux process.	[69]
	sRAGE overexpression (compared with control)	↑ Cell viability ↓ LDH cytotoxicity	↓ GSDMD‐NT ↓ Caspase 4	N/A	↑ LC3 II/I ↓ p62		
NRVMs cells	H/R (H: 2 h, R: 24 h)	N/A	↑ NLRP3 ↑ Caspase 1 ↑ GSDMD	↑ IL‐1β ↑ IL‐18	↑ TUNEL‐positive nuclei ↑ cTnI	H/R‐mediated cardiomyocyte injuries, pyroptosis and apoptosis in cultured primary cardiomyocytes under H/R insults.	[70]
	Beclin‐1 overexpression (compared with control)	N/A	↓ NLRP3 ↓ Caspase 1 ↓ GSDMD	↓ IL‐1β ↓ IL‐18	↓ TUNEL‐positive nuclei ↓ cTnI		
H9c2 cells	H/R (H: 2 h, R: 24 h)	↓ Cell viability	↑ NLRP3 ↑ ASC ↑ Caspase 1 ↑ Cleaved caspase 1 ↑ GSDMD ↑ GSDMD‐NT	↑ IL‐1β ↑ IL‐18	↓ ERP44 ↑ IP3R1 ↑ Maximal mitochondrial Ca^2+^ ↑ Maximal intracellular Ca^2+^	The expression of ERP44 was downregulated and IP3R1 was overexpressed leading to Ca^2+^ overload and NLRP3‐mediated pyroptosis in cardiomyocyte under H/R insults. Knockout of IP3R1 or overexpression of ERP44 or treatment with calcium channel blocker Nifedipine D6 alleviated Ca^2+^ overload, inflammation, and pyroptosis in cardiomyocyte under H/R insults. Treatment with NLRP3 activator Nigericin abolished the protective effects of ERP44 against Ca^2+^ overload and cardiomyocyte pyroptosis under H/R insults.	[55]
	IP3R1 knockdown (compared with control)	↑ Cell viability	↓ NLRP3 ↓ ASC ↓ Caspase 1 ↓ Cleaved caspase 1 ↓ GSDMD ↓ GSDMD‐NT	↓ IL‐1β ↓ IL‐18	↔ ERP44 ↓ IP3R1 ↓ Maximal mitochondrial Ca^2+^ ↓ Maximal intracellular Ca^2+^		
	IP3R1 knockdown + co‐treatment with Nigericin 10 μM under H/R condition (compared with IP3R1 knockdown)	↓ Cell viability	↑ NLRP3 ↑ ASC ↑ Caspase 1 ↑ Cleaved caspase 1 ↑ GSDMD ↑ GSDMD‐NT	N/A	N/A		
	ERP44 overexpression (compared with control)	↑ Cell viability	↓ NLRP3 ↓ ASC ↓ Caspase 1 ↓ Cleaved caspase 1 ↓ GSDMD ↓ GSDMD‐NT	↓ IL‐1β ↓ IL‐18	↓ Maximal mitochondrial Ca^2+^ ↓ Maximal intracellular Ca^2+^		
	ERP44 overexpression + co‐treatment with Nigericin 10 μM under H/R condition (compared with ERP44 overexpression)	↓ Cell viability	↑ NLRP3 ↑ ASC ↑ Caspase 1 ↑ Cleaved caspase 1 ↑ GSDMD ↑ GSDMD‐NT	N/A	N/A		
	Co‐treatment with Nifedipine D6 50 μM under H/R condition (compared with control)	↑ Cell viability	↓ NLRP3 ↓ ASC ↓ Caspase 1 ↓ Cleaved caspase 1 ↓ GSDMD ↓ GSDMD‐NT	↓ IL‐1β ↓ IL‐18	↑ ERP44 ↓ Maximal mitochondrial Ca^2+^ ↓ Maximal intracellular Ca^2+^		
NRVMs cells	H/R (H: 3 h, R: 2 h) (compared with control)	↓ Cell viability ↑ LDH cytotoxicity	↓ TNXIP ↑ NLRP3 ↑ ASC ↑ Cleaved caspase1 ↑ GSDM‐NT	↑ IL‐1β ↑ IL‐18	↑ p‐AMPK/AMPK ↑ p‐ACC/ACC ↓ SOD	H/R not only augmented the phosphorylation of AMPK and ACC but also diminished the antioxidative enzyme expression leading to NLRP3‐mediated pyroptosis‐related proteins.	[47]
H9c2 cells	H/R (H: 4, R: 2 h) (compared with control)	↓ Cell viability	↑ NLRP3 ↑ ASC ↑ Cleaved caspase 1 ↑ GSDMD	↑ IL‐1β ↑ IL‐18	↑ TUNEL‐positive nuclei	H/R augmented the expressions of NLRP3‐mediated pyroptosis‐related proteins and apoptosis in cardiomyocytes.	[71]
H9c2 cells	H/R (H: 4, R: 3 h) (compared with control)	↓ Cell viability ↑ LDH cytotoxicity	↑ NLRP3 ↑ Cleaved caspase 1 ↔ GSDMD ↑ GSDMD‐NT	↑ IL‐1β ↑ TNF‐α ↑ IL‐6	↑ TUNEL‐positive nuclei ↑ CK‐MB ↑ LncRNA PVT1	H/R‐induced LncRNA PVT1 overexpression leading to cardiomyocyte apoptosis and pyroptosis.	[59]
	LncRNA PVT1 knockdown (compared with wild type control)	↑ Cell viability ↓ LDH cytotoxicity	↓ NLRP3 ↓ Cleaved caspase 1 ↔ GSDMD ↓ GSDMD‐NT	↓ IL‐1β ↓ TNF‐α ↓ IL‐6	↓ TUNEL‐positive nuclei ↓ CK‐MB ↓ LncRNA PVT1	Knockdown of LncRNA PVT1 alleviated H/R‐induced cardiomyocyte apoptosis and pyroptosis.	
	LncRNA PVT1 overexpression (compared with wild type control)	↓ Cell viability	↔ GSDMD ↑ GSDMD‐NT	↑ IL‐1β	↑ LncRNA PVT1	Overexpression of LncRNA PVT1 aggravated H/R‐mediated cardiomyocyte pyroptosis.	
HL‐1 cells	H/R (H: 4, R: 4 h) (compared with control)	↓ Cell viability	↑ NLRP3 ↑ ASC ↔ Caspase 1 ↑ Cleaved caspase 1	↑ IL‐1β ↑ IL‐18	↑ LncRNA Neat1	H/R‐induced LncRNA Neat1overexpression leading to cardiomyocyte pyroptosis.	[72]
H9c2 cells	H/R (H: 4, R: 12 h) (compared with control)	↓ Cell viability ↑ LDH cytotoxicity	↑ NLRP3 ↑ ASC ↑ Caspase 1 ↑ Cleaved caspase 1 ↑ GSDMD ↑ GSDMD‐NT ↑ %Pyroptosis	↑ IL‐1β ↑ IL‐18	↓ cFLIP_L_	Overexpression of cFLIP_L_ alleviated H/R‐mediated cardiomyocyte pyroptosis.	[73]
	cFLIP_L_ overexpression (compared with control)	↑ Cell viability ↓ LDH cytotoxicity	↓ NLRP3 ↓ ASC ↑ Caspase 1 ↓ Cleaved caspase1 ↓ GSDMD ↓ GSDMD‐NT ↓ %Pyroptosis	↓ IL‐1β ↓ IL‐18	↑ cFLIP_L_		
H9c2 cells	H/R (H: 6 h, R: 1 h)	↓ Cell viability ↑ LDH cytotoxicity	↑ NLRP3 ↑ GSDMD‐NT	↑ IL‐1β	N/A	H/R‐induced cleavage of GSDMD‐mediated pyroptosis in cardiomyocytes and macrophages in H/R insults.	[16]
J774a.1 cells	H/R (H: 6 h, R: 1 h)	↓ Cell viability ↑ LDH cytotoxicity	↑ NLRP3 ↑ GSDMD‐NT	N/A	N/A		
Ana‐1 cells	H/R (H: 6 h, R: 1 h)	↓ Cell viability ↑ LDH cytotoxicity	↑ NLRP3 ↑ GSDMD‐NT	N/A	N/A		
Ana‐1 cells	H/R (H: 6 h, R: 1 h) Cotreatment with H_2_O_2_ 100 or 200 μM along with hypoxia	↓ Cell viability (dose dependently) ↑ LDH cytotoxicity (dose dependently)	↑ NLRP3 (only with 200 μM) ↑ GSDMD (only with 200 μM) ↑ GSDMD‐NT (only with 200 μM)	↑ IL‐1β (only with 200 μM)	N/A	Oxidative stress aggravated macrophages pyroptosis in H/R insults.	[16]
H9c2 cells	H/R (H: 6 h, R: 18 h)	↓ Cell viability ↑ LDH cytotoxicity	↑ NLRP3 ↑ Caspase 1 ↑ GSDMD‐NT ↑ %Pyroptosis	↑ IL‐1β ↑ IL‐18 ↑ TNF‐α ↑ IL‐6	↑ USP11 ↑ TRAF3 ↑ pIKKβ/IKKβ ↑ pNFκB/NF‐κB	H/R mediated the upregulation of USP11 thus prevented TRAF3 deubiquitination leading to cardiomyocyte pyroptosis. USP11 knockdown prevented H/R‐mediated TRAF3 overexpression and pyroptosis in cardiomyocyte/TRAF3 overexpression abrogated the protective effects of USP11 knockdown on H9C2 cells in H/R insults.	[74]
	USP11 knockdown (compared with control)	↑ Cell viability ↓ LDH cytotoxicity	↓ NLRP3 ↓ Caspase 1 ↓ GSDMD‐NT ↓ %Pyroptosis	↓ IL‐1β ↓ IL‐18 ↓ TNF‐α ↓ IL‐6	↓ USP11 ↓ TRAF3 ↓ pIKKβ/IKKβ ↓ pNFκB/NF‐κB		
	USP11 knockdown + TRAF3 overexpression (compared with control)	↓ Cell viability ↑ LDH cytotoxicity	↑ %Pyroptosis	↑ IL‐1β ↑ IL‐18 ↑ TNF‐α ↑ IL‐6	↔ USP11 ↑ TRAF3		
HL‐1 cells	H/R (H: 8 h, R: 12 h)	↓ Cell proliferation ↑ LDH cytotoxicity	↑ NLRP3 ↑ ASC ↑ Caspase 1 ↑ Cleaved caspase 1 ↑ GSDMD ↑ GSDMD‐NT	↑ IL‐1β	↑ ROS ↓ ΔΨm ↑ HIF‐1α ↑ TUG1	H/R‐induced HIF‐1α‐mediated TUG1 overexpression, cardiac mitochondrial dysfunction and pyroptosis. H/R treatment‐induced mitochondrial dysfunction and myocardial pyroptosis were inhibited by silencing TUG1 in cardiomyocytes.	[44]
	TUG1 knockdown (compared with control)	N/A	↓ NLRP3 ↓ ASC ↓ Caspase 1 ↓ Cleaved caspase 1 ↓ GSDMD ↓ GSDMD‐NT	↓ IL‐1β	↓ ROS ↑ ΔΨm ↓ FUS		
NMCMs cells	H/R (H: 8 h, R: 12 h)	↓ Cell proliferation ↑ LDH cytotoxicity	↑ NLRP3 ↑ ASC ↑ Caspase 1 ↑ Cleaved caspase 1 ↑ GSDMD ↑ GSDMD‐NT	↑ IL‐1β	↑ ROS ↓ ΔΨm ↑ HIF‐1α ↑ TUG1		
	TUG1 knockdown (compared with control)	N/A	↓ NLRP3 ↓ ASC ↓ Caspase 1 ↓ Cleaved caspase 1 ↓ GSDMD ↓ GSDMD‐NT	↓ IL‐1β	↓ ROS ↑ ΔΨm ↓ FUS		
HL‐1 cells	H/R (H: 8 h, R: 24 h)	↓ Cell viability ↑ LDH cytotoxicity	↑ AIM2 ↑ ASC ↑ Cleaved caspase 1 ↑ GSDMD‐NT	↑ IL‐1β ↑ IL‐18	↑ Lnc RNA MEG3 ↔ TAF15	H/R‐induced LncRNA MEG3/TAF15/AIM2‐mediated cardiomyocyte pyroptosis.	[60]
	MEG3 knockdown (compared with control)	↑ Cell viability ↓ LDH cytotoxicity	↓ AIM2 ↓ ASC ↓ Cleaved caspase 1 ↓ GSDMD‐NT	↓ IL‐1β ↓ IL‐18	↓ Lnc RNA MEG3		
H9c2 cells	H/R (H: 16 h, R: 2 h)	↑ LDH cytotoxicity	↑ NLRP3 ↑ ASC ↔ Caspase 1 ↑ Cleaved caspase 1 ↑ GSDMD	↑ IL‐1β ↑ IL‐18	↑ PI‐positive nuclei ↑ MDA ↓ Mitofilin ↓ p‐PI3K ↓ p‐AKT	Mitofilin was decreased in H/R cardiomyocyte and knockdown of mitofilin exacerbated H/R‐induced cardiomyocyte pyroptosis through PI3K/AKT signalling pathway. Caspase 1 inhibitor AC‐YVAD‐CMK mitigated the effect of mitofilin deprivation in cardiomyocyte under H/R condition.	[46]
	Mitofilin knockdown (compared with control)	↑ LDH cytotoxicity	↑ NLRP3 ↑ ASC ↔ Caspase 1 ↑ Cleaved caspase 1 ↑ GSDMD	↑ IL‐1β ↑ IL‐18	↑ PI‐positive nuclei ↑ MDA ↓ Mitofilin ↓ p‐PI3K ↓ p‐AKT		
	Mitofilin knockdown + cotreatment with AC‐YVAD‐CMK 10 (10 μg/mL) (compared with Mitofilin knockdown)	↓ LDH cytotoxicity	↓ NLRP3 ↓ ASC ↔ Caspase 1 ↓ Cleaved caspase 1 ↓ GSDMD	↓ IL‐1β ↓ IL‐18	↓ PI‐positive nuclei ↓ MDA ↔ Mitofilin		
	Mitofilin overexpression (compared with control)	↓ LDH cytotoxicity	↓ NLRP3 ↓ ASC ↔ Caspase 1 ↓ Cleaved caspase 1 ↓ GSDMD	↓ IL‐1β ↓ IL‐18	↓ PI‐positive nuclei ↓ MDA ↑ p‐PI3K ↑ p‐AKT	Mitofilin protected against H/R‐induced cardiomyocyte pyroptosis through PI3K/AKT‐dependent signalling pathway.	
	Mitofilin overexpression + cotreatment with GDC‐0941 5 μM (compared with control)	↑ LDH cytotoxicity	↑ NLRP3 ↑ ASC ↔ Caspase 1 ↑ Cleaved caspase 1 ↑ GSDMD	↑ IL‐1β ↑ IL‐18	↑ MDA		
NMCMs cells	H/R (H: 24 h, R: 12 h)	↓ Cell viability ↓ Cell survival	↑ NLRP3 ↑ ASC ↑ Caspase 1 ↑ Cleaved caspase 1 ↑ GSDMD ↑ GSDMD‐NT	↑ IL‐1β ↑ IL‐18	↑ LDH ↑ ROS	H/R‐induced GSDMD‐mediated cardiomyocyte pyroptosis. GSDMD was the key determination of cardiomyocyte pyroptosis activation under H/R insults.	[45]
	GSDMD overexpression (compared with control)	↓ Cell viability ↓ Cell survival	↑ NLRP3 ↑ ASC ↑ Caspase 1 ↑ Cleaved caspase 1 ↑ GSDMD ↑ GSDMD‐NT	↑ IL‐1β ↑ IL‐18	↑ LDH ↑ ROS		
	GSDMD knockdown (compared with control)	↑ Cell viability ↑ Cell survival	↓ NLRP3 ↓ ASC ↓ Caspase 1 ↓ Cleaved caspase 1 ↓ GSDMD ↓ GSDMD‐NT	↓ IL‐1β ↓ IL‐18	↓ LDH ↓ ROS		
H9c2 cells	Hypoxia 2 h	↑ LDH cytotoxicity	↑ NLRP3 ↑ Caspase 1 ↑ Cleaved caspase 1 ↑ GSDMD‐NT	↑ IL‐1β ↑ IL‐18	↑ TLR4 ↑ p65	Hypoxia caused NLRP3 inflammasome‐mediated cardiomyocyte pyroptosis through the activation of the TLR4/NF‐κB/p65 signalling pathway.	[75]
H9c2 cells	Hypoxia 6 h LncRNA H19 overexpression (compared with control)	N/A	↓ NLRP3 ↓ ASC ↓ Caspase 1	↓ IL‐1β ↓ IL‐18	↓ TUNEL‐positive nuclei ↑ LncRNA H19 ↑ CyclinD1, PCNA	LncRNA H19 overexpression suppressed pyroptosis activation in cardiomyocytes under hypoxic condition. Interfering of LncRNA H19 expression aggravated pyroptosis activation in cardiomyocytes under hypoxic condition.	[67]
	LncRNA H19 knockdown (compared with control)	N/A	↑ NLRP3 ↑ ASC ↑ Caspase 1	↑ IL‐1β ↑ IL‐18	↑ TUNEL‐positive nuclei ↓ LncRNA H19 ↓ CyclinD1, PCNA		
	CYP1B1 overexpression (compared with control)	N/A	↑ NLRP3 ↑ ASC ↑ Caspase 1	↑ IL‐1β ↑ IL‐18	↑ TUNEL‐positive nuclei ↑ CYP1B1 ↓ CyclinD1, PCNA	CYP1B1 overexpression aggravated pyroptosis activation in cardiomyocytes under hypoxic condition. Interfering of CYP1B1 expression mitigated pyroptosis activation in cardiomyocytes under hypoxic condition.	
	CYP1B1 knockdown (compared with control)	N/A	↓ NLRP3 ↓ ASC ↓ Caspase 1	↓ IL‐1β ↓ IL‐18	↓ TUNEL‐positive nuclei ↑ CYP1B1 ↑ CyclinD1, PCNA		
	LncRNA H19 overexpression + CYP1B1 overexpression (compared with CYP1B1 overexpression)	N/A	↓ NLRP3 ↓ ASC ↓ Caspase 1	↓ IL‐1β ↓ IL‐18	↓ TUNEL‐positive nuclei ↑ CYP1B1 ↑ CyclinD1, PCNA	LncRNA H19 regulated pyroptosis of cardiomyocytes through inhibition of CYP1B1.	
NMVMs cells	Hypoxia 6 h	↓ Cell viability	↑ NLRP3 ↑ ASC ↑ Caspase1 ↑ Cleaved caspase 1 ↑ GSDMD ↑ GSDMD‐NT	↑ IL‐18	↑ LDH ↑ PI‐positive nuclei ↑ RBP4 ↑ MDA, ROS ↓ GSH, SOD	RBP4 regulates cardiomyocyte pyroptosis via interaction with NLRP3 in hypoxic cardiomyocyte. Knockdown of RPB4 attenuated cardiomyocyte pyroptosis through inhibition of NLRP3 signalling pathway in hypoxic cardiomyocyte. Treatment with NLRP3 inhibitor MCC950 protected against hypoxia‐induced cardiomyocyte pyroptosis in hypoxic cardiomyocyte.	[48]
	RPB4 knockdown (compared with control)	↑ Cell viability	↓ NLRP3 ↓ ASC ↓ Caspase1 ↓ Cleaved caspase 1 ↓ GSDMD ↓ GSDMD‐NT	↓ IL‐18	↓ LDH ↓ PI‐positive nuclei ↓ RBP4		
	RPB4 overexpression (compared with control)	↓ Cell viability	↑ NLRP3 ↑ ASC ↑ Caspase 1 ↑ Cleaved caspase 1 ↑ GSDMD ↑ GSDMD‐NT	↑ IL‐18	↑ LDH ↑ PI‐positive nuclei ↑ RBP4		
	RPB4 overexpression + NLRP3 knockdown (compared with control)	↑ Cell viability	↓ NLRP3 ↓ ASC ↓ Caspase 1 ↓ Cleaved caspase 1 ↓ GSDMD ↓ GSDMD‐NT	↓ IL‐18	↓ LDH ↓ PI‐positive nuclei ↔ RBP4		
	RPB4 overexpression + MCC950 10 μM (compared with control)	↑ Cell viability	↓ NLRP3 ↓ ASC ↓ Caspase 1 ↓ Cleaved caspase 1 ↓ GSDMD ↓ GSDMD‐NT	↓ IL‐18	↓ LDH ↓ PI‐positive nuclei ↔ RBP4		
H9c2 cells	Hypoxia 12 h	↓ Cell viability	↑ NLRP3 ↑ Cleaved caspase 1 ↑ GSDMD‐NT	N/A	↑ LDH ↑ PI‐positive nuclei	Hypoxia‐induced GSDMD‐mediated cardiomyocyte pyroptosis.	[76]
H9c2 cells	Hypoxia + TNF‐α 40 ng/mL 12 h (compared with control)	↓ Cell viability ↑ LDH cytotoxicity	↑ NLRP3 ↑ Cleaved caspase 1 ↑ GSDMD‐NT	N/A	↓ SIRT1 ↑ NOX4 ↑ PI‐positive nuclei	Hypoxia and TNF‐α‐mediated cardiomyocyte pyroptosis through SIRT1/NOX4/ROS signalling pathway.	[77]
NMCMs cells	Hypoxia 12 h	↓ Cell viability	↑ NLRP3 ↑ ASC ↑ Cleaved caspase 1 ↑ GSDMD‐NT ↑ IL‐18	↑ IL‐1β	↓ GDF11 mRNA ↓ GDF11 protein ↓ HOXA3 protein	GDF11 was abnormally downregulated during hypoxic condition leading to pyroptosis in hypoxic cardiomyocytes. GDF11 overexpression inhibits cardiomyocytes pyroptosis via HOXA3/ NLRP3 signalling pathway in hypoxic cardiomyocytes.	[78]
	GDF11 overexpression (compared with control)	↑ Cell viability	↓ NLRP3 ↓ ASC ↓ Cleaved caspase 1 ↓ GSDMD‐NT ↓ IL‐18	↓ IL‐1β	↑ HOXA3 protein		
NRCMs cells	Hypoxia 12 h	↓ Cell viability ↑ LDH cytotoxicity	↑ NLRP3 ↑ ASC ↑ Cleaved caspase 1 ↑ GSDMD‐NT	↑ IL‐1β	N/A	Hypoxia‐induced GSDMD‐mediated pyroptosis in rat cardiomyocytes.	[79]
NMCMs cells	Hypoxia 24 h	↓ Cell viability	↑ NLRP3 ↑ Cleaved caspase 1 ↑ GSDMD ↑ GSDMD‐NT	↑ IL‐1β ↑ IL‐18	↑ TUNEL‐positive nuclei ↑ PI‐positive nuclei	Hypoxia‐induced GSDMD‐mediated pyroptosis and apoptosis in mouse cardiomyocytes.	[80]
NMCMs cells	Hypoxia 24 h	↓ Cell viability ↑ LDH cytotoxicity	↑ NLRP3 ↑ Cleaved caspase 1 ↑ GSDMD‐NT	↑ IL‐1β ↑ IL‐18	↑ PI‐positive nuclei ↓ LncRNA FAF	Hypoxia‐induced downregulation of LncRNA FAF and enhanced GSDMD‐mediated pyroptosis in mouse cardiomyocyte. Overexpression of LncRNA FAF mitigated hypoxia‐induced cardiomyocyte pyroptosis. Interfering with lncRNA FAF expression aggravated hypoxia‐induced cardiomyocyte pyroptosis.	[61]
	Adv‐FAF overexpression (compared with control)	↑ Cell viability ↓ LDH cytotoxicity	↓ NLRP3 ↓ Cleaved caspase 1 ↓ GSDMD‐NT	↓ IL‐1β ↓ IL‐18	↓PI‐positive nuclei ↑ LncRNA FAF		
	si‐FAF knockdown (compared with control)	↓ Cell viability ↑ LDH cytotoxicity	↑ NLRP3 ↑ Cleaved caspase 1 ↑ GSDMD‐NT	↑ IL‐1β ↑ IL‐18	↑ PI‐positive nuclei ↓ LncRNA FAF		
NMCMs cells	Hypoxia 48 h	↓ Cell viability	↑ GSDMD ↑ GSDMD‐NT	↑ IL‐1β ↑ IL‐18	↑ LDH ↑ PCSK9 ↑ mtDNA damage ↑ mtROS	PCSK9 regulated Caspase 1‐dependent pyroptosis through mtDNA damage and mitochondrial oxidative stress in hypoxic cardiomyocyte.	[56]
	PCSK9 overexpression (compared with control)	↓ Cell viability	↑ GSDMD ↑ GSDMD‐NT	↑ IL‐1β ↑ IL‐18	↑ LDH ↑ mtDNA damage ↑ mtROS		
	PCSK9 knockdown (compared with control)	↑ Cell viability	↓ GSDMD ↓ GSDMD‐NT	↑ IL‐1β ↑ IL‐18	↓ LDH ↓ mtDNA damage ↓ mtROS		
H9c2 cells	OGD 12 or 24 h	↓ Cell viability	↑ NLRP3 ↑ Cleaved caspase 1 ↑ GSDMD‐NT	↑ IL‐1β ↑ IL‐18	↑ HMGB1	OGD induced GSDMD‐mediated pyroptosis in rat cardiomyocytes.	[81]
H9c2 cells	OGD 6, 12, 24, 48 h	N/A	↑ Cleaved caspase 1 ↑ GSDMD ↑ GSDMD‐NT	↑ IL‐1β ↑ IL‐18	↑ IRF2 (time dependently) ↑ HIF1 (time dependently) ↑ Apoptosis	OGD mediated IRF2 overexpression through HIF1 signalling thus promoted GSDMD‐mediated pyroptosis.	[82]
	IRF2 silencing (compared with control)	N/A	↓ Cleaved caspase 1 ↓ GSDMD ↓ GSDMD‐NT	↑ IL‐1β ↑ IL‐18	↓ IRF2 ↓ Apoptosis		

## 4 Evidence of Canonical Pyroptosis in IHD: Reports Across In Vivo Studies

In addition to research studies performed in vitro, pyroptosis has also been demonstrated in animal models of IHD. Pyroptosis was detected in rats and mice that underwent coronary microembolisation (CME)‐induced microinfarction [77, 83]. CME initiated canonical pyroptosis via the TLR4/NF‐κB/NLRP3/caspase 1 signalling pathway, resulting in cardiac injuries, inflammation and impaired cardiac function in the rat and mouse models [77, 83]. During I/R, AMI and post‐MI, there was a significant escalation in the expression of canonical pyroptosis components (i.e., NLRP3, ASC, cleaved caspase 1 and GSDMD‐NT) [45, 48, 56, 74, 79, 80]. The activation of caspase 1 and of GSDMD resulted in the secretion of IL‐1β and IL‐18 [17, 69, 74, 79, 80]. IHD‐mimicking insults in mice and rats have been observed to cause the development of systolic and diastolic dysfunction, exacerbate cardiac geometry abnormalities, ultimately leading to heart failure and increase mortality rates [45, 48, 56, 74, 79, 80].

We have recently reported that GSDMD‐mediated pyroptosis emerged as the predominant programmed cell death mechanism in post‐MI pathology [18]. More interestingly, and unlike the other pyroptosis‐related pathologies, only GSDMD but not gasdermin E (GSDME) was found to be specifically responsible for pyroptosis in the context of IHD [17]. In contrast to canonical and noncanonical pyroptosis, the alternative pathway of pyroptosis did not appear to be involved in IHD pathogenesis. Supporting this, the crucial significance of GSDMD‐dependent pyroptosis in IHDs was definitively established through genetic manipulation in experiments, where the absence of GSDMD markedly attenuated pyroptosis activation, reduced cardiac fibrosis and injuries, consequently resulting in the preservation of systolic function in I/R mice [16, 17]. In addition, the impairment of autophagy flux augmented caspase 4 and GSDMD led to myocardial pyroptosis in cardiac I/R mice [70]. However, enhancing autophagy by Beclin‐1 overexpression could attenuate caspase 4‐dependent pyroptosis in I/R mice [70].

Consistent with the cellular studies, the findings from the in vivo experiments consistently supported the association between cardiac mitochondria and myocardial pyroptosis in IHD. Cardiac I/R aggravates both cardiac and mitochondrial Ca^2+^ overload, thereby triggering NLRP3‐mediated pyroptosis in rats [55]. These studies added weight to the findings of a previous study which reported mitochondrial dysfunction and an imbalance in mitochondrial dynamics contributed to post‐MI‐induced pyroptosis and subsequent heart failure [18]. However, investigations regarding alterations in mitochondrial functions and mitochondrial dynamic control and their relationships with cardiac pyroptosis in the context of ischaemic insults are underexplored and warrant further investigation. This collective evidence underscores the intricate involvement of cardiac mitochondria in the pathophysiological cascade, leading to the processes associated with pyroptosis and consequential cardiac complications in the context of IHD.

Taken together, the reports across in vitro and in vivo experiments suggest that exposure to hypoxic, ischaemic and reperfusion insults in the heart is associated with the activation of canonical and noncanonical pyroptosis pathways rather than an alternative pathway. This in turn leads to the loss of functional cardiomyocytes and causes myocardial injury, pathological remodelling and ventricular dysfunction, ultimately contributing to the development of heart failure [45, 48, 56, 74, 79, 80]. The comprehensively summarised key features pertinent to canonical pyroptosis in the in vivo experiments are shown in Table 2.

**TABLE 2:** Pyroptosis in IHD models: reports across the in vivo studies.

Study models	Methods	Major findings				Interpretation	Reference
		Functional outcome	Pyroptosis	Inflammation	Other biochemical markers		
SD rats	Coronary microembolisation by polyethylene microspheres intraventricular injection for 6 h	↓ %LVEF, %LVFS ↓ CO ↑ LVEDd	↑ NLRP3 ↑ ASC ↑ Caspase 1	↑ IL‐1β ↑ IL‐18 ↑ TNF‐α	↑ %Microinfarction ↑ Serum cTnI ↑ TLR4 ↑ MyD88 ↑ NF‐κB p65	CME‐induced myocardial pyroptosis, cardiac injuries and inflammation through TLR4/Myd88/NF‐κB signalling pathway in rats.	[83]
C57BL/6 mice	Coronary microembolisation by polyethylene microspheres intraventricular injection for 72 h	↓ %LVEF, %LVFS	↑ NLRP3 ↑ Cleaved caspase 1 ↑ GSDMD‐NT	↑ IL‐1β	↑ %Microinfarction ↑ LDH ↑ Collagen content	CME‐induced myocardial pyroptosis, cardiac injuries and dysfunction in mice.	[76]
C57BL/6 mice	Cardiac I/R (I: 30 min, R: 1 h)	N/A	↑ NLRP3 ↑ ASC ↔ Caspase 1 ↑ Cleaved caspase 1 ↔ GSDMD ↑ GSDMD‐NT	↑ IL‐1β ↑ IL‐18	↑ %Infarction ↑ LDH ↑ cTnT ↑ CK‐MB ↑ LncRNA Neat1	LncRNA Neat1 was increased in cardiac I/R and leading to myocardial pyroptosis and inflammation in I/R rats.	[72]
SD rats	Cardiac I/R (I: 30 min, R: 2 h)	N/A	↑ NLRP3 ↑ Caspase1 ↑ GSDMD‐NT	↑ IL‐1β ↑ IL‐18 ↑ TNF‐α ↑ IL‐6	↑ %Infarction ↑ LDH ↑ TUNEL‐positive nuclei ↑ Cardiomyocyte size ↑ USP11 ↑ TRAF3 ↑ pIKKβ/IKKβ ↑ pNFκB/NF‐κB	USP11 was increased in cardiac I/R and caused the upregulation of TRAF3, leading to myocardial pyroptosis and inflammation in I/R rats. The inhibition of USP11 improved post‐I/R injury by increased TRAF3 ubiquitination thus reduced myocardial pyroptosis and inflammation in I/R rats.	[74]
	USP11 knockdown (compared with control)	N/A	↓ NLRP3 ↓ Caspase1 ↓ GSDMD‐NT	↓ IL‐1β ↓ IL‐18 ↓ TNF‐α ↓ IL‐6	↓ %Infarction ↓ LDH ↓ TUNEL‐positive nuclei ↓ Cardiomyocyte size ↓ USP11 ↓ TRAF3 ↓ pIKKβ/IKKβ ↓ pNFκB/NF‐κB		
SD rats	Cardiac I/R (I: 30 min, R: 2 h)	N/A	↑ NLRP3 ↑ ASC ↑ Cleaved caspase 1	↑ IL‐1β ↑ IL‐18	↑ %Infarction ↑ cTnI ↑ CK‐MB ↑ TUNEL‐positive nuclei ↑ FoxO3a ↑ ARC	Cardiac I/R‐mediated myocardial injury via the activation of miR‐29b‐activated FoxO3a/ARC axis in rats.	[84]
SD rats	Cardiac I/R (I: 30 min, R: 2 h)	N/A	↑ NLRP3 ↑ ASC ↑ Caspase 1 ↑ Cleaved caspase 1 ↓ GSDMD ↑ GSDMD‐NT	↑ IL‐1β ↑ IL‐18	↑ %Infarction ↑ LDH ↑ CK‐MB ↓ cFLIP_L_	Overexpression of cFLIP_L_ alleviated I/R‐induced myocardial pyroptosis in rats.	[73]
	cFLIP_L_ overexpression (compared with control)	N/A	↓ NLRP3 ↓ ASC ↓ Caspase 1 ↓ Cleaved caspase 1 ↑ GSDMD ↓ GSDMD‐NT	↓ IL‐1β ↓ IL‐18	↓ %Infarction ↓ LDH ↓ CK‐MB ↑ cFLIP_L_		
C57BL/6 mice	Cardiac I/R (I: 30 min, R: 3 h)	↓ %LVEF, %LVFS ↑ LVEDd, LVESd	↑ NLRP3 ↑ ASC ↑ Caspase 1 ↑ Cleaved caspase 1 ↑ GSDMD ↑ GSDMD‐NT	↑ IL‐1β ↑ IL‐18	↑ %Infarction ↑ LDH ↑ TUNEL‐positive nuclei ↑ ROS ↓ miRNA‐182‐5p	Cardiac I/R‐blunted miRNA‐182‐5p expression, augmented ROS, cardiac injuries, and GSDMD‐mediated myocardial pyroptosis in mice.	[45]
C57BL/6 mice	Cardiac I/R (I: 30 min, R: 24 h)	N/A	↑ Caspase 11 ↑ Cleaved caspase 11 ↑ GSDMD ↑ GSDMD‐NT ↔ GSDME	N/A	↑ IS/AAR ↔ AAR/LV ↑ Serum LDH	Cardiac I/R‐mediated myocardial injury via Cleaved caspase 11/GSDMD‐ but not GSDME‐mediated pyroptosis in mice.	[17]
	Cardiomyocyte specific GSDMD knockout (compared with wild type)	N/A	↓ GSDMD ↑ GSDMD‐NT	N/A	↓ IS/AAR ↔ AAR/LV ↓ Serum LDH		
C57BL/6 mice	Cardiac I/R (I: 30 min, R: 24 h)	↓ %LVEF, %LVFS ↓ CO, SV	↑ NLRP3 ↑ Caspase 1 ↑ GSDMD	↑ IL‐1β ↑ IL‐18 ↑ p‐IκBα/IκBα ↑ p‐NF‐κB p65/NF‐κB p65	↑ %Infarction ↑ cTnI ↑ TUNEL‐positive nuclei	Cardiac I/R augmented GSDMD‐mediated myocardial pyroptosis through NF‐κB pathway in mice. sRAGE prevented GSDMD‐mediated myocardial pyroptosis inhibited IκBα/NF‐κB p65 signalling thus improve cardiac function in I/R mice.	[69]
	sRAGE overexpression (compared with wild type)	↑ %LVEF, %LVFS ↑ CO, SV	↓ NLRP3 ↓ Caspase 1 ↓ GSDMD	↓ IL‐1β ↓ IL‐18 ↓ p‐IκBα/IκBα ↓ p‐NF‐κB p65/NF‐κB p65	↓ %Infarction ↓ cTnI ↓ TUNEL‐positive nuclei		
C57BL/6 mice	Cardiac I/R (I: 45 min, R: 2 h, 24 h, 3 days, and 7 days)	↓ %LVEF, %LVFS	↑ IL‐6 ↑ GSDMD (only on Day 3) ↑ GSDMD‐NT (only on Day 1)	↑ IL‐1β ↑ IL‐18	↑ IS/AAR, AAR/LV	Cleavage of GSDMD‐NT was augmented in the myocardium leading to pyroptosis, cardiac inflammation and cardiac dysfunction in I/R mice. GSDMD deficiency alleviated the inflammatory response by regulating pyroptosis, reduced the infarct size and preserved cardiac function in cardiac I/R mice.	[16]
	GSDMD knockout (compared with wild type)	↑ %LVEF, %LVFS	↓ GSDMD	↓ IL‐1β ↓ IL‐18 ↓ IL‐6 ↓ inflammatory response‐related genes	↓ IS/AAR ↔ AAR/LV ↓ apoptosis‐related genes		
C57BL/6 J mice	Cardiac I/R (I: 45 min, R: 1, 6, and 12 h)	↓ %Survival	↑ GSDMD‐NT ↑ Caspase 4	↑ CD11b‐positive cells ↑ F40/80 positive cells	↑ IS/LV and AAR/LV ↑ Serum LDH ↑ Serum CK ↑ Beclin‐1, LC3 II/I, p62	The impairment of autophagy flux augmented caspase 4 and GSDMD expression leading to myocardial pyroptosis in cardiac I/R mice. Beclin‐1 overexpression attenuates myocardial pyroptosis in cardiac I/R mice.	[70]
	Beclin‐1 overexpression (compared with wild type)	↑ %Survival	↓ GSDMD‐NT ↓ Caspase 4	↓ CD11b‐positive cells ↓ F40/80 positive cells	↓ IS/LV, ↑ AAR/LV ↓ Serum LDH ↓ Serum CK ↓ Beclin‐1, LC3 II/I, p62		
SD rats	Cardiac I/R (I: 45 min, R: 2 h)	↓ LVEDP ↓ dP/dt(max) ↑ dP/dt(min)	↑ NLRP3 ↑ ASC ↑ Caspase 1 ↑ GSDMD	↓ COX I ↑ COX II ↑ TNF‐α ↑ IL‐6	↑ %Infarction ↑ cTnT	Cardia I/R‐induced cardiac dysfunction by augmenting myocardial inflammation and pyroptosis.	[85]
C57BL/6J mice	Cardiac I/R (I: 45 min, R: 4 h)	↓ %LVEF, %LVFS ↑ LVEDd, LVESd	↑ NLRP3 ↑ ASC ↑ Cleaved caspase 1 ↑ Caspase 1 activity ↑ GSDMD	↑ IL‐1β ↑ IL‐18	↑ %Infarction ↑ TUNEL‐positive nuclei ↑ LDH ↑ CK ↑ CK‐MB ↑ p‐AMPK/AMPK ↑ p‐ACC/ACC ↑ ROS ↓ SOD	Cardiac I/R not only augmented the phosphorylation of AMPK and ACC but also induced pyroptosis activation thus causing myocardial injuries and cardiac dysfunctions in mice.	[47]
C57BL/6 mice	Cardiac I/R (I: 45 min, R: 48 h)	N/A	↑ NLRP3 ↑ Cleaved caspase 1 ↔ GSDMD ↑ GSDMD‐NT	↑ IL‐1β ↑ TNF‐α ↑ IL‐6	↑ LDH ↑ cTnI ↑ BNP ↑ CK‐MB ↑ LncRNA PVT1 ↓ α‐MHC ↑ β‐MHC ↑ TLR4 ↑ MyD88 ↑ p‐p65/p65 ↑ Bax ↓ Bcl‐2 ↑ Cleaved caspase 3	I/R‐mediated myocardial damage and inflammation through the upregulation of LncRNA PVT1 in the myocardial tissues leading to the activation of pyroptosis via TLR4/MyD88/NF‐κB/NLRP3 inflammasome pathway. Silencing of PVT1 alleviates myocardial damage, cardiac inflammation and pyroptosis activation through the inhibition of TLR4/MyD88/NF‐κB/NLRP3 signalling pathway in I/R mice.	[59]
	PVT1 knockdown (compared with wild type)	N/A	↓ NLRP3 ↓ Cleaved caspase 1 ↔ GSDMD ↓ GSDMD‐NT	↓ IL‐1β ↓ TNF‐α ↓ IL‐6	↓ LDH ↓ cTnI ↓ BNP ↓ CK‐MB ↓ LncRNA PVT1 ↑ α‐MHC ↓ β‐MHC ↓ TLR4 ↓ MyD88 ↓ p‐p65/p65 ↓ Bax ↑ Bcl‐2 ↓ Cleaved caspase 3		
SD rats	Cardiac I/R (I: 30 min, R: 72 h)	N/A	↑ NLRP3 ↑ ASC ↑ Caspase 1 ↑ Cleaved caspase 1 ↑ GSDMD ↑ GSDMD‐NT	↑ IL‐1β ↑ IL‐18	↑ IS/AAR ↔ AAR/LV ↑ LDH ↑ CK‐MB ↓ ERP44 ↑ IP3R1 ↑ Maximal mitochondrial Ca^2+^ ↑ Maximal intracellular Ca^2+^	Cardiac I/R aggravated ERP44‐IP3R1‐mediated Ca^2+^ overload and NLRP3‐mediated pyroptosis in rats. Downregulation of IP3R1 attenuated cardiac I/R‐induced myocardial injury by reduced calcium overload and NLRP3‐mediated pyroptosis.	[55]
	IP3R1 knockdown (compared with wild type)	N/A	↓ NLRP3 ↓ ASC ↓ Caspase 1 ↓ Cleaved caspase 1 ↓ GSDMD ↓ GSDMD‐NT	↓ IL‐1β ↓ IL‐18	↓ IS/AAR ↔ AAR/LV ↓ LDH ↓ CK‐MB ↔ ERP44 ↓ IP3R1 ↓ Maximal mitochondrial Ca^2+^ ↓ Maximal intracellular Ca^2+^		
Wistar rats	Cardiac I/R (I: 45 min, R: 72 h)	N/A	↑ Cleaved caspase 1 ↑ GSDMD ↑ GSDMD‐NT	↑ IL‐1β ↑ Cleaved IL‐1β ↑ TNF‐α ↑ CRP ↓ IL‐10	↑ %Infarction ↑ TUNEL‐positive nuclei ↑ LDH	Cardiac I/R‐induced myocardial pyroptosis, apoptosis, cardiac injuries and inflammation.	[86]
SD rats	Cardiac I/R (I: 1 h, R: 2 h)	↓ %LVEF, %LVFS	↑ NLRP3 ↑ Cleaved caspase 1/Caspase 1 ↑ GSDMD‐NT/GSDMD	↑ IL‐1β ↑ IL‐18 ↑ IL‐6 ↑ TNF‐α	↑ IS/AAR ↑ cTnT ↑ LDH ↑ CK‐MB ↑ TUNEL‐positive nuclei ↑ Incidence of VF ↑ Duration of VF ↑ OGDHL ↑ ROS	Cardiac I/R‐induced myocardial pyroptosis, apoptosis, cardiac injuries, inflammation and ventricular fibrillation in mice.	[87]
C57BL/6 mice	Cardiac I/R (I: 1 h, R: 24 h)	N/A	↑ NLRP3 ↑ ASC ↑ Caspase 1 ↑ Cleaved caspase 1 ↑ GSDMD ↑ GSDMD‐NT	↑ IL‐1β	↑ %Infarction ↑ LDH ↑ CK ↑ CK‐MB ↑ TUNEL‐positive nuclei ↑ ROS ↑ TUG1	HIF‐1α regulated TUG1 overexpression leading to mitochondrial dysfunction and myocardial pyroptosis in cardiac I/R mice. TUG1 silencing ameliorated cardiac I/R‐induced mitochondrial dysfunction and myocardial pyroptosis.	[44]
	TUG1 knockdown (compared with wildtype)	N/A	↓ NLRP3 ↓ ASC ↓ Caspase 1 ↓ Cleaved caspase 1 ↓ GSDMD ↓ GSDMD‐NT	↓ IL‐1β	↓ %Infarction ↓ LDH ↓ CK ↓ CK‐MB ↓ TUNEL‐positive nuclei ↓ ROS ↓ TUG1		
C57BL/6 mice	Permanent LAD ligation for 12 h	↓ %LVEF, %LVFS ↑ LVIDd, LVIDs	↑ NLRP3 ↑ ASC ↑ Cleaved caspase 1 ↑ GSDMD‐NT	↑ IL‐1β ↑ IL‐18	↓ GDF11 mRNA ↓ GDF11 protein ↓ HOXA3 mRNA ↓ HOXA3 protein	GDF11 was abnormally downregulated thus leading to myocardial pyroptosis in AMI mice. GDF11 inhibits myocardial pyroptosis via HOXA3/ NLRP3 signalling pathway in AMI mice.	[78]
	AAV9‐GDF11 overexpression (compared with wildtype)	↑ %LVEF, %LVFS ↓ LVIDd, LVIDs	↓ NLRP3 ↓ ASC ↓ Cleaved caspase 1 ↓ GSDMD‐NT	↓ IL‐1β ↓ IL‐18	↑ HOXA3 protein		
C57BL/6 mice	Permanent LAD ligation for 12 h	N/A	↑ NLRP3 ↑ ASC ↔ Caspase 1 ↑ Cleaved caspase 1 ↑ GSDMD	↑ IL‐1β ↑ IL‐18	↑ %Infarction ↑ LDH ↑ MDA ↓ Mitofilin	AMI‐caused the reduction of mitofilin and upregulation of GSDMD‐mediated pyroptosis in mice. Overexpression of mitofilin‐mitigated GSDMD‐mediated pyroptosis in AMI mice.	[46]
	AAV9‐Mitofilin overexpression (compared with wildtype)	N/A	↓ NLRP3 ↓ ASC ↔ Caspase 1 ↓ Cleaved caspase 1 ↓ GSDMD	↓ IL‐1β ↓ IL‐18	↓ %Infarction ↓ LDH ↓ MDA ↑ Mitofilin		
C57BL/6 mice	Permanent LAD ligation for 24 h	↓ %LVEF, %LVFS	↑ NLRP3 ↑ Cleaved caspase 1 ↑ GSDMD ↑ GSDMD‐NT	↑ IL‐1β ↑ IL‐18	↑ %Infarction ↑ LDH	AMI‐induced GSDMD‐mediated pyroptosis in mice leading to myocardial injury and cardiac dysfunction in mice.	[80]
C57BL/6 mice	Permanent LAD ligation for 24 h	↓ %LVEF, %LVFS	↑ NLRP3 ↑ Cleaved caspase 1 ↑ GSDMD‐NT	↑ IL‐1β ↑ IL‐18	↑ %Infarction ↑ LDH	AMI‐induced GSDMD‐mediated pyroptosis in mice leading to myocardial injury and cardiac dysfunction in mice.	[75]
Wistar rats	Permanent LAD ligation for 24 h	↓ %LVEF, %LVFS ↑ LVIDd, LVIDs	↑ NLRP3 ↑ ASC ↑ Cleaved caspase 1 ↑ GSDMD	↑ IL‐1β ↑ Inflammatory cells infiltration	↑ %Infarction ↑ Serum cTnI, BNP ↑ 8‐OHdG‐positive cells ↑ ROS ↑ MDA ↑ TUNEL‐positive nuclei	AMI‐induced cardiac oxidative stress mediated pyroptosis and inflammation leading to cardiac dysfunction in rats.	[88]
C57BL/6 mice	Permanent LAD ligation for 72 h	↓ %LVEF	↑ Caspase1 mRNA (at 6 h after AMI) ↑ Caspase1 activity (at 1 h after AMI) ↑ GSDMD activity (at 1 h after AMI)	↑ CD4 T cell ↑ CD8 T cell ↑ Monocyte ↑ Macrophage	↑ %Infarction	Caspase 1 activity significantly increased at 1 h after AMI leading to GSDMD‐mediated pyroptotic overactivation, inflammatory infiltration and cardiac dysfunction in AMI mice.	[19]
SD rats	Permanent LAD ligation for 72 h	↓ %LVEF, %LVFS ↑ LVEDd, LVESd	↑ NLRP3 ↑ ASC ↑ Cleaved caspase 1 ↔ GSDMD ↑ GSDMD‐NT	↑ IL‐1β ↑ IL‐18	↑ %Infarction ↑ Serum cTnI, LDH ↑ Myocardial ATP, ADP, AMP ↑ TLR4 ↑ MyD88 ↑ NF‐κB p65	AMI‐induced myocardial pyroptosis through TLR4/MyD88/NF‐κB/NLRP3 signalling pathway leading to myocardial injuries, cardiac inflammation and cardiac dysfunction in rats.	[23]
C57BL/6 mice	Permanent LAD ligation for 72 h	↓ %LVEF, %LVFS	↑ Cleaved caspase 1 ↑ GSDMD ↑ GSDMD‐NT	↑ IL‐1β	↑ IRF2 ↑ TUNEL‐positive nuclei	AMI‐induced IRF2 overexpression and GSDMD‐NT mediated pyroptosis in mice. IRF2 silencing attenuates GSDMD‐NT‐mediated pyroptosis in AMI mice.	[82]
	IRF2 silencing (compared with wildtype)	↑ %LVEF, %LVFS	↓ Cleaved caspase 1 ↓ GSDMD ↓ GSDMD‐NT	↓ IL‐1β	↓ TUNEL‐positive nuclei		
SD rats	Permanent LAD ligation for 1 week	N/A	↑ NLRP3 ↑ ASC ↑ Caspase1 ↑ Cleaved caspase 1	N/A	↑ LDH	AMI‐induced myocardial pyroptosis in rats.	[89]
C57BL/6 mice	Permanent LAD ligation for 1 week	N/A	↑ NLRP3 ↑ ASC ↑ Caspase1 ↑ GSDMD ↑ Serum GSDMD	↑ IL‐1β ↑ IL‐18	↑ LDH	MI‐induced GSDMD‐mediated myocardial pyroptosis in mice. Downregulation of PCSK9 inhibited pyroptosis in serum and suppressed GSDMD‐mediated myocardial pyroptosis in mice after MI.	[56]
	PCSK9 knockout (compared with wildtype)	N/A	↓ NLRP3 ↓ ASC ↓ Caspase1 ↓ GSDMD ↓ Serum GSDMD	↓ IL‐1β ↓ IL‐18	↓ LDH		
C57BL/6 mice	Permanent LAD ligation for 1 week	↓ %LVEF, %LVFS	↑ NLRP3 ↑ ASC ↑ Caspase1 ↑ Cleaved caspase 1 ↑ GSDMD ↑ Serum GSDMD	↑ IL‐1β ↑ IL‐18	↑ %Infarction ↑ LDH ↑ MDA, ROS ↓ GSH, SOD ↑ RBP4 (at Days 1 and 3 after AMI) ↓RBP4 (at Day 7 after AMI)	RBP4 promotes cardiac injury and dysfunction by inducing cardiomyocyte pyroptosis through an interaction with NLRP3 in AMI mice. Downregulation of RBP4‐mitigated cardiac pyroptosis, cardiac injuries and dysfunction by inhibiting with NLRP3 activation in AMI mice.	[48]
	RBP4 knockout immediately after LAD ligation (compared with wildtype)	↑ %LVEF, %LVFS ↓ LVIDd, LVIDs	↓ NLRP3 ↓ ASC ↓ Caspase1 ↓ Cleaved caspase 1 ↓ GSDMD ↓ GSDMD‐NT	↓ IL‐1β ↓ IL‐18	↓ %Infarction ↓ ANP, BNP, MYH7 ↓ LDH ↓ PI‐positive nuclei ↓ RBP4		
SD rats	Permanent LAD ligation for 1 week	↓ %LVEF, %LVFS	↑ NLRP3 ↑ Cleaved caspase 1 ↑ GSDMD‐NT	↑ IL‐1β ↑ IL‐18	↑ %Infarction ↓ LncRNA FAF	LncRNA FAF was downregulated leading to myocardial pyroptosis in AMI rats. Overexpression of LncRNA FAF improved cardiac function and suppressed pyroptosis in AMI rats.	[61]
	Adv‐FAF overexpression after LAD ligation (compared with wildtype)	↑ %LVEF, %LVFS	↓ NLRP3 ↓ Cleaved caspase 1 ↓ GSDMD‐NT	↓ IL‐1β ↓ IL‐18	↓ %Infarction		
SD rats	Permanent LAD ligation for 1 week	↓ %survival ↓ %LVEF ↑ LVEDD	↑ NLRP3 ↑ ASC ↑ Caspase 1	N/A	↑ %Infarction ↑ TUNEL positive nuclei ↓ LncRNA H19 ↑ CYPB1	LncRNA H19 were downregulated and caused CYP1B1 overexpression leading to myocardial pyroptosis and cardiac dysfunction in AMI rats. LncRNA H19 overexpression or CYP1B1 knockdown attenuates MI progression and myocardial pyroptosis in AMI rats.	[67]
	H19 overexpression after LAD ligation (compared with wildtype)	N/A	↓ NLRP3 ↓ ASC ↓ Caspase 1	N/A	↓ %Infarction ↓ TUNEL positive nuclei ↑ LncRNA H19 ↓ CYPB1		
	CYPB1 knockdown after LAD ligation (compared with wildtype)	N/A	↓ NLRP3 ↓ ASC ↓ Caspase 1	N/A	↓ %Infarction ↓ TUNEL positive nuclei ↔ LncRNA H19 ↑ CYPB		
SD rats	Permanent LAD ligation for 1 week	↓ %LVEF, %LVFS ↑ LVIDd, LVIDs	↑ NLRP3 ↑ Caspase1 ↑ Cleaved caspase 1 ↑ GSDMD	↑ IL‐1β ↑ IL‐18	↑ %Infarction ↑ %Collagen volume fraction ↑ TAK1 ↑ p‐JNK	MI‐induced GSDMD‐mediated pyroptosis and associated with the TAK1/JNK pathway leading to cardiac dysfunction in AMI rats.	[90]
C57BL/6 J mice	Permanent LAD ligation for 4 weeks	↓ %survival ↓ %LVEF	↑ NLRP3 ↑ Cleaved caspase 1 ↑ GSDMD‐NT	↑ IL‐1β ↑ Cleaved IL‐1β ↑ IL‐18 ↑ Cleaved IL‐18	↑ %Fibrosis ↑ Cardiomyocyte size ↑ ROS ↑ α‐SMA ↑ Collagen I	MI‐induced myocardial fibrosis and cardiac remodelling by inhibiting ROS/Caspase‐1/GSDMD‐NT signalling pathway after MI.	[91]
C57BL/6 J mice	Permanent LAD ligation for 4 weeks	↓ %LVEF, %LVFS	↑ AIM2 ↑ ASC ↑ NLRP3 ↑ Cleaved caspase 1 ↑ GSDMD‐NT	↑ IL‐1β ↑ IL‐18	↑ %Fibrosis ↑ LDH ↑ CK‐MB	MI‐induced LncRNA MEG3/TAF15/AIM2‐mediated pyroptosis leading to cardiac dysfunction after MI.	[60]
	MEG3 knockdown (compared with wild type)	↑ %LVEF, %LVFS	↓ AIM2 ↓ ASC ↓ NLRP3 ↓ Cleaved caspase 1 ↓ GSDMD‐NT	↓ IL‐1β ↓ IL‐18	↓ %Fibrosis ↓ LDH ↓ CK‐MB		
FVB/NJ mice	Permanent LAD ligation for 4 weeks	↓ %LVEF, %LVFS ↑ LVIDd, LVIDs ↑ LVEDd, LVEDs	↑ CLMP mRNA (time dependently) ↑ CLMP protein ↑ Cleaved caspase 1 ↑ GSDMD‐NT	↑ IL‐1β ↑ C4b ↑ Ccl8	↑ %Fibrosis ↑ LDH ↑ Myeloperoxidase ↑ Ly6g	Clmp deficiency aggravated myocardial pyroptosis, trigger more severe inflammation response and myocardial injury after MI.	[92]
	Clmp knockdown (compared with wild type)	↓ %LVEF, %LVFS ↑ LVIDd, LVIDs ↑ LVEDd, LVEDs	↓ CLMP mRNA (time dependently) ↓ CLMP protein ↑ Cleaved caspase 1 ↑ GSDMD‐NT	↑ IL‐1β ↑ C4b ↑ Ccl8	↑ %Fibrosis ↑ LDH ↑ Myeloperoxidase ↑ Ly6g		
SD rats	Permanent LAD ligation for 4 weeks	↓ %survival ↓ %LVEF, %LVFS ↑ LVIDd, LVIDs	↑ NLRP3 ↑ ASC ↑ Caspase 1 ↑ GSDMD	↑ IL‐1β	↑ %Fibrosis ↑ Plasma BNP ↑ Cardiac tissue MDA ↑ α‐SMA ↑ Collagen type I and III	MI‐aggravated cardiac pyroptosis, cardiac fibroblasts proliferation and activation leading to adverse cardiac remodelling and cardiac dysfunction in rats.	[79]
Wistar rats	Permanent LAD ligation for 5 weeks	↓ %LVEF ↓ E/A ratio ↓ LVESP ↑ LVEDP ↑ LVEDV, LVESP ↓ SV ↓ CO	↑ NLRP3 ↑ GSDMD ↑ GSDMD‐NT	N/A	↑ Serum cTnT, NT‐proBNP ↑ Serum MDA ↑ Cardiac tissue MDA ↑ HW/BW ratio ↑ Lung W/D weight ↑ Cleaved caspase 3/Caspase 3 ↑ pMLKL/tMLKL ↔ ACSL4 ↑ Mitochondrial dysfunctions ↑ pDRP1/tDRP1 ↓ MFN1 ↓ OPA1	MI worsened cardiac mitochondrial function, mitochondrial dynamic imbalance, apoptosis, necroptosis and pyroptosis but not ferroptosis. GSDMD‐mediated pyroptosis was the most dominant programmed cell death in post‐MI pathology.	[18]

## 5 IHD‐Mediated Oxidative Stress Caused Pyroptosis Through the Noncanonical Pathway: Reports From In Vitro and In Vivo Studies

In addition to the caspase 1‐mediated GSDMD pathway (canonical pyroptosis), IHD has been implicated in the initiation of pyroptosis through the caspase 1‐independent pathway or noncanonical pyroptosis. Quite separately to caspase 1, caspases 4, 5 and 11 have also been reported to be correlated with pyroptosis in the context of IHD pathologies. Recently, Sun et al. [70] demonstrated that H/R caused markedly increased oxidative stress and triggered caspase 4 inflammasome activation in cultured cardiac endotheliocytes. H/R has also been shown to exacerbate autophagy flux impairment, which subsequently led to caspase 4‐dependent pyroptosis [70]. Subsequently, promotion of autophagy via Beclin‐1 overexpression was found to potentially reverse these detrimental effects [70]. In line with the in vitro findings, their results from animal experiments further confirmed the escalation of ROS‐induced caspase 4/GSDMD‐mediated pyroptosis in I/R mice [70]. Moreover, a recent study by Shi et al. [17] reported that H/R and cardiac I/R insults led to a significant surge in oxidative stress. Oxidative stress and oxidised phospholipid then triggered the activation of caspase 11 (caspases 4 and 5 in humans), subsequently causing caspase 11/GSDMD‐mediated pyroptosis [17, 93].

**FIGURE 2:**
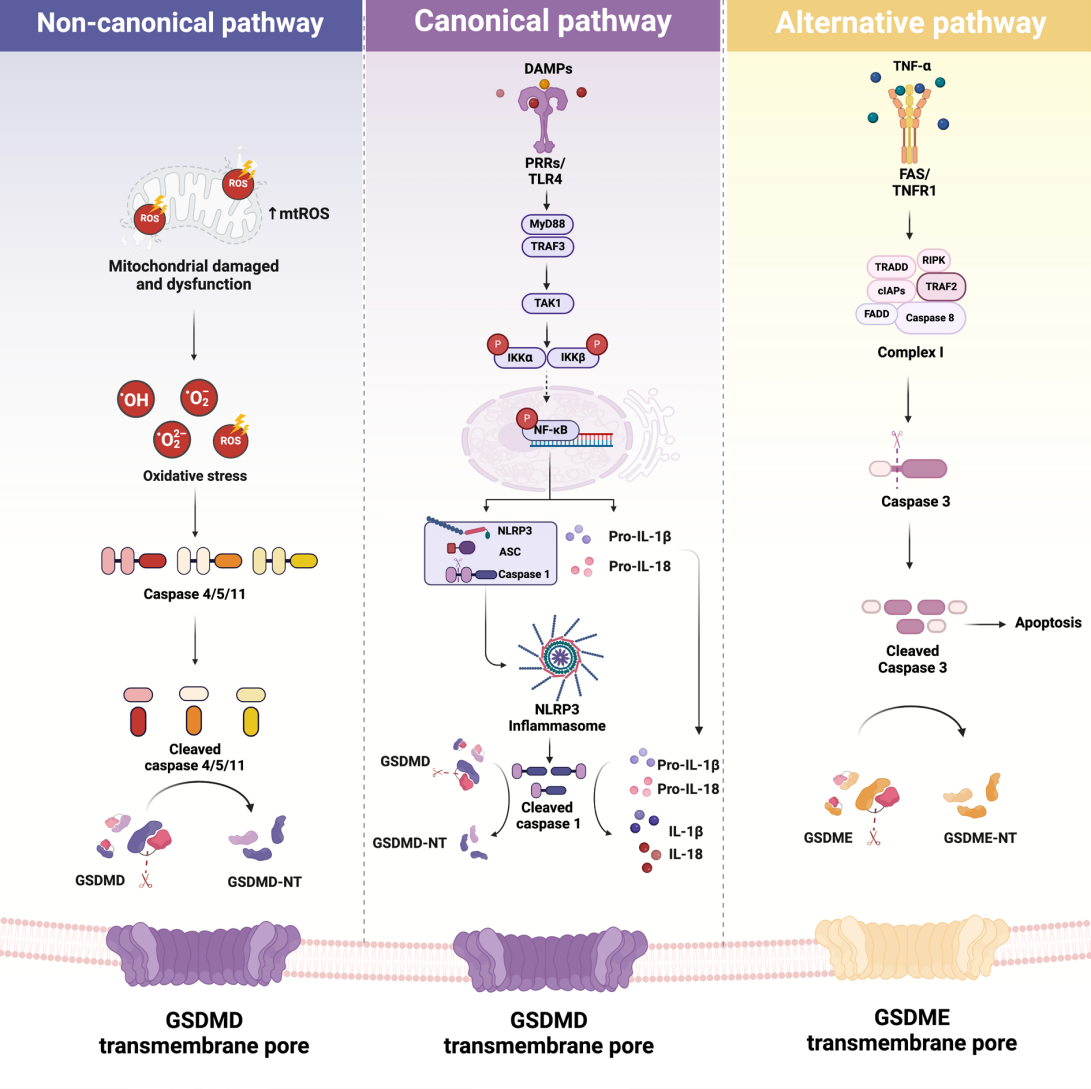
IHD‐mediated pyroptosis occurs through canonical, noncanonical and alternative pathways. In the case of canonical pyroptosis, during IHD, several DAMPs bind to their respective PRRs, leading to the activation of NF‐κB/NLRP3/caspase 1/GSDMD‐mediated pyroptosis. The presence of mtROS and mtDNA produced by damaged and dysfunctional mitochondria facilitates the assembly of the inflammasome and subsequent activation of caspase 1, leading to canonical pyroptosis. In noncanonical pyroptosis, procaspases 4, 5 and 11 are activated through the escalation of ROS during I/R or H/R. This activation subsequently leads to the cleavage of GSDMD, thereby mediating noncanonical pyroptosis. The alternative pathway of pyroptosis is triggered by the binding and activation of TNFR1 with TNF‐α, which in turn activates caspase 3. The proteolytic actions of cleaved caspase 3 not only induced apoptosis but also initiated GSDME‐mediated pyroptosis. However, in association with the pathogenesis of IHDs, only canonical and noncanonical pyroptosis pathways were observed, with no evidence of alternative pyroptosis. DAMPs, damage‐associated molecular patterns; GSDMD, gasdermin D; GSDMD‐NT, *N*‐terminal domain of gasdermin D; GSDME, gasdermin E; GSDME‐NT, *N*‐terminal domain of gasdermin E; IL‐18, interleukin 18; IL‐1β, interleukin 1β; mtDNA, mitochondrial DNA; mtROS, mitochondrial ROS; NF‐κB, nuclear factor kappa B; NLRP3, nucleotide‐binding domain, leucine‐rich‐containing family, pyrin domain‐containing‐3; PAMPs, pathogen‐associated molecular patterns; TNFR1, tumour necrosis factor receptor 1; TNF‐α, tumour necrosis factor alpha.

The activation of caspase 4/5/11 has also been shown to cleave pannexin‐1, resulting in ATP release, potassium efflux, and NLRP3 activation [94]. Collectively, IHD, in particular H/R and cardiac I/R insults, not only induced NLRP3/caspase 1/GSDMD activation, leading to conventional pyroptosis activation, but also intensified oxidative stress. This heightened oxidative stress, in turn, triggered noncanonical pyroptosis via the oligomerisation and activation of caspase 4/5/11. The understanding concerning the involvement of noncanonical pyroptosis in the setting of IHD, although extensive, is currently insufficient due to very limited evidence and needs further validation. The key features used for differentiation between canonical and noncanonical pyroptosis in IHD are illustrated in Figure 2. It has been reported that various cell death modalities, including apoptosis, necroptosis, ferroptosis, pyroptosis and autophagy‐related death, concurrently participate in the pathogenesis of different types of IHD [50, 51, 52, 53]. Apart from canonical and noncanonical pyroptosis, previous studies also reported the coexistence of other PCD mechanisms in IHD pathologies. In cellular studies, hypoxic and H/R insults not only mediated canonical and noncanonical pyroptosis but also exacerbated autophagy flux impairment and apoptosis, reducing cell viability in cultured cardiomyoblasts and cardiac microvascular endothelial cells [44, 54, 55, 56, 57, 58]. In animal studies, the coexistence of pyroptosis with autophagy‐related cell death, apoptosis, ferroptosis and necroptosis has been observed in settings such as AMI, cardiac I/R injury and post‐MI‐induced heart failure [35, 44, 59, 60, 61, 62]. These findings underscore the complexity of cell death mechanisms in IHD, highlighting the interplay between pyroptosis and other PCD pathways, which may collectively contribute to disease progression and present potential therapeutic targets for mitigating cardiac injury.

## 6 Evidence of Canonical Pyroptosis in IHD: Reports Across Clinical Studies

Although extensive investigation into the role of pyroptosis in IHD through preclinical research has been reported, only a few clinical investigations have demonstrated its significant contribution to the pathology in patients with IHD. Several reports have shown significantly higher levels of immunologically determined GSDMD molecules in the serum of patients with AMI, STEMI and CMI in comparison to those in individuals without IHD [16, 17, 56]. These findings suggest that GSDMD could serve as a promising biomarker and therapeutic target for identifying and intervening in myocardial pyroptosis in IHD patients. Proinflammatory markers such as IL‐1β and IL‐18 have also been found to be consistently elevated in the serum of IHD patients [56]. This underscores the significance of pyroptosis in the pathophysiology of IHD in clinical settings and suggest that targeting pyroptotic pathways could be a novel therapeutic strategy against conditions associated with IHD.

Given the novel nature of the research into pyroptosis in cardiovascular disease, particularly in the context of IHD, the majority of recent studies have been conducted in vitro and in vivo. Consequently, only a limited number of studies have provided evidence of pyroptosis in clinical settings. However, Wang and colleagues reported that the expression of GSDMD in peripheral blood mononuclear cells (PBMCs) was found to be heightened in AMI patients with single or multivessel disease following coronary angiography or percutaneous coronary intervention, indicating a close association with AMI‐related pyroptosis [98]. The receiver operating characteristic (ROC) analysis of relevant data also demonstrated that the GSDMD level exhibited substantial diagnostic predictability for AMI (95% CI 0.791–0.941 in single vessel disease and 95% CI 0.926–0.991 in multivessel disease) [98]. Likewise, Shi et al. [17] also verified higher levels of GSDMD in the serum of AMI patients 1 h after PCI. More specifically, Ye et al. [16] reported that serum levels of GSDMD and IL‐18 were notably increased in AMI patients only after, but not before PCI. Additionally, the study by Wang et al. also reported a notable increase in PCSK9, NLPR3, caspase 1, IL‐1β, IL‐18, and GSDMD in the serum of post‐MI patients in comparison to the age‐matched nondisease group [26]. However, GSDMD exhibited specific lipid binding abilities, particularly with cardiolipin, phosphatidylethanolamine, phosphatidylinositol phosphates, and phosphatidylserine, which are specifically situated in the inner leaflet of the plasma membrane [15, 34]. Hence, the lipid specificity ensured that the circulating GSDMD released from pyroptotic cells would not trigger pyroptosis in neighbouring cells [15, 34]. These findings suggest that GSDMD could serve as a promising biomarker for prognostic and diagnostic tools for assessing myocardial pyroptosis in IHD patients. Proinflammatory markers such as IL‐1β and IL‐18 have also been consistently found to be elevated in the serum of IHD patients [56].

In addition, several studies have also documented an association between inflammasome priming and activation in a clinical context. Previous studies had reported the heightened concentrations of NLRP3 and its downstream signalling, including caspase 1, pro‐IL‐1β, IL‐11β, pro‐IL‐18 and IL‐18, in the peripheral blood PBMCs of AMI patients [95, 96]. Wang et al. [96] also found that the levels of NLRP3 have a positive correlation with atherosclerosis severity (Gensini score), indicating that the existence of the NLRP3 inflammasome cascade is relevant to the severity of both AMI and coronary atherosclerosis. A recent study by Mo et al. [97] reported that plasma levels of the NLRP3 inflammasome were increased in AMI patients. It was also observed that the heightened levels of the inflammasome showed a correlation with increased B‐type natriuretic peptides (BNP) and a reduction of the left ventricular ejection fraction (LVEF) [97]. Taken together, accumulating evidence from recent clinical studies suggests that the detection of the NLRP3 inflammasome and relevant pyroptosis proteins could offer valuable insights for clinical prognosis and be considered a potential therapeutic target for patients with IHD. Nevertheless, owing to the current scarcity of clinical studies, additional research is warranted to gain a better understanding of the precise mechanisms involved and to explore potential interventions aimed at mitigating pyroptosis‐related pathologies in IHD patients. All of these clinical reports are summarised in Table 3.

**TABLE 3:** Pyroptosis in IHD models: reports across the clinical studies.

Study models	Methods	Major findings				Interpretation	Reference
		Functional outcome	Pyroptosis	Inflammation	Other biochemical markers		
Patient with AMI	PBMCs isolated from peripheral venous blood from: Patient with single vessel AMI (*n* = 40) Patient with multivessel AMI (*n* = 60) (compared with healthy control)	↓ %LVEF ↓ LVEDD	↑ Serum GSDMD	↑ Serum CRP	↑ Serum LDL ↑ Serum TC ↑ WBC	GSDMD expression level in PBMCs was elevated in AMI patients and was closely associated with the pyroptosis of AMI.	[98]
Patient with STEMI	Peripheral venous blood samples (*n* = 19) (compared with stable CAD)	N/A	↑ Serum GSDMD after PCI for 1 h	N/A	N/A	GSDMD levels were increased in the serum of STEMI patients after 1 h of PCI.	[17]
Patient with STEMI	Peripheral venous blood samples (*n* = 29) (compared with healthy control)	N/A	↔ Serum GSDMD before PCI ↑ Serum GSDMD after PCI	↔ Serum IL‐18 before PCI ↑ Serum IL‐18 after PCI	N/A	Serum levels of GSDMD and IL‐18 is increased in STEMI patients after PCI.	[16]
Patient with STEMI	Peripheral venous blood samples (*n* = 13) (compared with healthy control)	N/A	N/A	N/A	↓ Serum LncRNA H19 ↑ Serum CYP1B1	Downregulation of LncRNA H19 and upregulation of CYP1B1 expressions were observed in serum of AMI patients.	[67]
Patients with CMI	Peripheral venous blood samples (*n* = 21) (compared with healthy control)	N/A	↑ Serum NLRP3 ↑ Serum Caspase 1 ↑ Serum GSDMD	↑ Serum IL‐1β ↑ Serum IL‐18	↑ Serum LDH ↑ Serum PCSK9	Serum levels of PCSK9 and pyroptosis‐related protein were highly expressed in patients with CMI.	[56]

## 7 Modulation of Canonical Pyroptosis as a Potential Therapeutic Target in IHD: Reports From In Vitro Studies

Several interventions have been initially implemented to mitigate the progressive nature of pyroptosis in the in vitro models of IHD‐mimicking pathologies. Since pyroptosis is classically activated through the NLRP3/caspase 1/GSDMD axis, several studies have investigated the efficacy of specific inhibitors targeting this axis to mitigate pyroptotic death in IHD. MCC950 is a synthetic small molecule inhibitor that selectively inhibits the activation of NLRP3 inflammasomes [24]. Specifically, MCC950 directly binds with the Walker B motif within NLRP3 [99]. Consequently, NLRP3 loses its capability to maintain an active structural conformation, leading to the inhibition of NLRP3‐induced ASC oligomerisation and caspase 1 cleavage [24, 99]. As expected, in the context of IHD, MCC950 treatment effectively mitigated hypoxia‐induced cardiomyocyte pyroptosis [23]. In addition, the small molecule VX‐765 (Belnacasan) has been identified as a potent and selective caspase 1/4 inhibitor [100, 101]. Mechanistically, VX‐765 competitively binds to the catalytic cysteine residue of caspase 1/4, thereby inhibiting their catalytic activities [100]. In the heart, pretreatment with VX‐765 could prevent autophagy flux impairment‐induced pyroptosis in association with H/R insults [70]. In addition, the inhibition of caspase 4/1 using VX‐765 effectively attenuated cell death in TNF‐α‐treated H9c2 cells subjected to hypoxic conditions [76]. More recently, Zhong et al. [34] employed structure‐based virtual screening to identify a novel GSDMD inhibitor namely GI‐Y1. Their molecular docking findings revealed that GI‐Y1 specifically binds with GSDMD‐NT at Arg7, a crucial site for lipid membrane affinity, consequently mitigating the formation of transmembrane pores [34]. Pretreatment with GI‐Y1 effectively attenuated pyroptosis and enhanced viability in NRVM cells exposed to H/R insults [34]. In this regard, GI‐Y1 provided satisfactory cardioprotective effects against H/R‐induced cardiomyocyte pyroptosis through the direct inhibition of GSDMD‐NT [34]. Taken together, the application of specific inhibitors targeting the NLRP3/caspase 1/GSDMD axis posed promising therapeutic efficacy against canonical pyroptosis‐mediated IHD in the in vitro models.

Increasing evidence also indicates the spatiotemporal impact of ROS on NF‐κB‐mediated NLRP3 inflammasome activation [102, 103]. Considering that ROS emerged as a potent intrinsic stimulus contributing to pyroptosis, therapeutic potential through ROS modulation could serve as a viable approach for offering cardioprotection in IHD‐mediated pyroptosis [104, 105]. Similarly, pretreatment with *N*‐acetylcysteine (NAC) could mitigate NLRP3‐mediated pyroptosis by regulating mitochondrial ROS in hypoxic cardiomyocytes with TNF‐α stimulation [76]. Likewise, a previous study demonstrated that co‐treatment with NAC effectively attenuated oxidative stress, thereby mitigating p‐NF‐kB/NLRP3/GSDMD‐mediated pyroptosis in cardiomyocytes subjected to OGD conditions [89]. The same study further revealed that cotreatment with pyrrolidine dithiocarbamate, which exhibited radical scavenging properties, also effectively attenuated NLRP3/GSDMD‐mediated pyroptosis in cardiomyocytes under OGD conditions [89]. Moreover, Liraglutide, a glucagon‐like peptide‐1 (GLP‐1) receptor agonist, displayed antioxidant and anti‐inflammatory properties conferring cytoprotection by regulating the SIRT1/NOX4/ROS signalling pathway in hypoxic cardiomyocytes with TNF‐α stimulation [77]. It has also been reported that rosuvastatin, a widely used lipid‐lowering agent, could protect against NLRP3‐mediated pyroptosis by diminishing mtROS in hypoxic cardiomyocytes with TNF‐α stimulation [76]. Taken together, the application of antioxidation therapy exhibited promising therapeutic efficacy against NLRP3/GSDMD‐mediated pyroptosis in the in vitro models of IHD.

Recently, exosomes have been shown to have potentials as a therapeutic approach for IHDs due to their ability to carry bioactive molecules, such as proteins, RNA and lipids, which can modulate inflammation, promote myocardial repair and enhance myocardial regeneration [31, 32, 106]. Their targeted delivery and ability to facilitate intercellular communication make them promising candidates for improving outcomes in IHDs by promoting healing and reducing injury [32, 107]. Various sources of exosomes have been tested for their efficacy in addressing IHD pathologies, with mesenchymal stem cell‐derived exosomes (MSCs‐exo) being one of the most extensively studied. MSCs‐exo contained miR‐320b, miR‐100‐5p and miR182‐5p were shown to ameliorate pyroptosis activation via the mitigation of NLRP3 inflammasome activation thus improving cell viability in cultured cardiomyocytes and cardiac microvascular endothelial cells under H/R insults [33, 34, 35, 45, 108]. Another source of exosome such as M2 macrophage‐derived exosomes (M2‐exo) or cardiac fibroblast secreted exosomes (CFs‐exo) were also shown the promising antipyroptosis efficacy against H/R injuries [34, 36, 109, 110]. M2‐exo carried miR‐148a while CFs‐exo contained miR‐133a were shown to enhance cardiomyocyte viability by mitigating NLRP3‐mediated canonical pyroptosis under H/R conditions [34, 36]. Collectively, exosomes from various sources show promise in inhibiting NLRP3‐mediated canonical pyroptosis in H/R conditions and may hold potential for treating IHDs in the future.

Several herbal or natural extracts have also been reported as having antipyroptotic properties in preclinical experiments. Pretreatment with emodin, a natural anthraquinone derivative, alleviated oxidative stress and mitigated cardiomyocytes pyroptosis through the inhibition of the TLR4/MyD88/nF‐κB/NLRP3 inflammasome pathway in H/R cardiomyocytes [68]. Also, pretreatment with Geniposide, an iridoid glycoside, could induce AMPK activation and alleviate oxidative stress levels, thus mitigating NLRP3 inflammasome‐mediated pyroptosis in H/R insults [47]. A green tea extract, epigallocatechin gallate, has been shown to alleviate cardiomyocyte pyroptosis by inhibiting the long noncoding RNA MEG3 [60]. This inhibition effectively mitigated the caspase 1/GSDMD axis in cardiomyocytes subjected to H/R insults [60]. Ginsenoside Rh2, a natural bioactive product derived from ginseng, decreased the level of expression of the protein HMGB1 and reduced the occurrence of the NLRP3 inflammatory cascade, thus preventing pyroptosis activation in the cardiomyocytes under OGD conditions [81]. These findings indicated that several natural extracts effectively exerted cytoprotective effects mainly through the inhibition of the NLRP3 inflammasome and the reduction in the activity of the canonical pyroptosis pathway. All interventions targeting pyroptosis in the in vitro experiments are summarised in Table 4.

**TABLE 4:** Evidence of interventions on pyroptosis in IHD models: reports across in vitro studies.

Study models	Methods	Intervention	Major findings				Interpretation	Reference
			Cell viability/functional outcome	Pyroptosis	Inflammation	Other biochemical markers		
NRVMs cells	H/R (H: 1 h, R: 2 h)	Pretreatment with Emodin 5 or 10 mM 1 h prior to H/R	↓ LDH cytotoxicity	↓ NLRP3 ↓ ASC ↓ Cleaved caspase 1 ↔ GSDMD ↓ GSDMD‐NT	↓ IL‐1β	↓ TLR4 ↓ MyD88 ↓ p‐p65/p65 ↓ p‐iκBα/iκBα ↓ MDA ↓ DHE ↑ SOD	Pretreatment with Emodin alleviated oxidative stress and mitigated cardiomyocyte pyroptosis through the inhibition of Tlr4/MyD88/nF‐κB/NLRP3 inflammasome pathway.	[68]
HCMECs cells	H/R (H: 2 h, R: 2 h)	Pretreatment with VX‐765 10 μM 2 h prior to H/R (compared with control)	↓ LDH cytotoxicity	↓ GSDMD‐NT ↓ Caspase 4	N/A	↑ Beclin‐1, LC3 II/I ↓ p62	Pretreatment with caspase 1 inhibitor VX‐765 prevented autophagy flux impairment and pyroptosis activation in H/R insults.	[70]
NRVMs cells	H/R (H: 3 h, R: 2 h)	Pretreatment with Geniposide 40 μM 20 min prior to H/R (compared with control)	↑↑ Cell viability ↓↓ LDH cytotoxicity	↓↓ TNXIP ↓↓ NLRP3 ↓↓ ASC ↓↓ Cleaved caspase 1 ↓↓ GSDMD‐NT ↓↓ IL‐18	↓↓ IL‐1β	↑↑ p‐AMPK/AMPK ↑↑ p‐ACC/ACC ↑↑ SOD	Pretreatment with Geniposide‐induced AMPK activation, alleviated oxidative stress level thus mitigated NLRP3 inflammasome‐mediated pyroptosis in H/R insults. The AMPK‐inhibitor Compound C blunted the protective effects of Geniposide in an AMPK‐dependent manner.	[47]
		Pretreatment with Geniposide 40 μM + Compound C 20 μM 20 min prior to H/R (compared with control)	↑ Cell viability ↓ LDH cytotoxicity	↓ TNXIP ↓ NLRP3 ↓ ASC ↓ Cleaved caspase 1 ↓ GSDMD‐NT ↓ IL‐18	↓ IL‐1β	↑ p‐AMPK/AMPK ↑ p‐ACC/ACC ↑ SOD		
NRCMs cells	H/R (H: 2 h, R: 24 h)	Pretreatment with M2‐exosome 24 h prior to H/R (compared with control)	↑ Cell viability ↓ LDH cytotoxicity	↓ NLRP3 ↓ ASC	N/A	↑ miR‐148a ↓ CK ↓ CK‐MB ↓ TUNEL‐positive nuclei ↓ Intracellular Ca^2+^ ↓ IP3R ↓ SERCA2a ↓ TXNIB ↓ TLR4 ↓ MyD88 ↓ p‐NF‐κB p65/ NF‐κB p65 ↓ p‐lκBα/lκBα ↑ miR‐18a	M2‐exosome carried miR‐148a which ameliorated cardiomyocyte pyroptosis, injuries, intracellular Ca^2+^ overload via TXNIP/TLR4/MyD88/NF‐κB/NLRP3 signalling pathway under H/R insults.	[110]
H9c2 cells	H/R (H: 4, R: 2 h)	Pretreatment with Oxytocin 10 nM 1 h prior to H/R (compared with control)	↑ Cell viability	↓ NLRP3 ↓ ASC ↓ Cleaved caspase 1 ↓ GSDMD	↓ IL‐1β ↓ IL‐18	↓ TUNEL‐positive nuclei	Oxytocin preconditioning reduced the expressions of NLRP3 and pyroptosis‐related proteins in cardiomyocyte in both normoglycaemia and hyperglycaemia condition under H/R insults.	[71]
		Pretreatment with Oxytocin 10 nM 1 h prior to H/R in 33 mM glucose (compared with control)	↑ Cell viability	↓ NLRP3 ↓ ASC ↓ Cleaved caspase 1 ↓ GSDMD	↓ IL‐1β ↓ IL‐18	↑ TUNEL‐positive nuclei		
NMCMs cells	H/R (H: 4, R: 2 h)	Pretreatment with Soluble uric acid 100 mg/L 4 h prior to H/R (compared with control)	↓ Cell viability ↑ LDH cytotoxicity	↑ NLRP3 ↑ ASC ↑ Caspase1 ↑ GSDMD	↑ IL‐1β	↑ TUNEL‐positive nuclei ↑ ROS ↑ Caspase 3	Uric acid mitigated cardiomyocyte pyroptosis via ROS/NLRP3 pathway under H/R insults.	[111]
		Pretreatment with soluble uric acid 100 mg/L + Bay11–7082 5 μM 4 h prior to H/R (compared with control)	↑ Cell viability ↓ LDH cytotoxicity	↓ NLRP3 ↓ ASC ↓ Caspase 1 ↓ GSDMD	↓ IL‐1β	↓ TUNEL‐positive nuclei ↓ ROS ↓ Caspase 3	Bay11–7082 and N‐acetylcysteine mitigated cardiomyocyte pyroptosis via ROS/NLRP3 pathway under high uric acid and H/R insults.	
		Pretreatment with soluble uric acid 100 mg/L + *N*‐acetylcysteine 10 mmol/L 4 h prior to H/R (compared with control)	↑ Cell viability ↓ LDH cytotoxicity	↓ NLRP3 ↓ ASC ↓ Caspase 1 ↓ GSDMD	↓ IL‐1β	↓ TUNEL‐positive nuclei ↓ ROS ↓ Caspase 3		
HK‐1 cells	H/R (H: 4, R: 4 h)	Pretreatment with Chlorogenic acid 0.2 μM 12 h prior to H/R (compared with control)	↑ Cell viability	↓ NLRP3 ↓ ASC ↔ Caspase 1 ↓ Cleaved caspase 1 ↓ GSDMD‐NT	↓ IL‐1β ↓ IL‐18	↓ LncRNA Neat1	Chlorogenic acid inhibited Lnc Neat1/NLRP3 inflammasome‐mediated pyroptosis in H/R cardiomyocyte.	[72]
		Pretreatment with Chlorogenic acid 2 μM 3 h prior to H/R (compared with control)	↑ Cell viability	↓ NLRP3 ↓ ASC ↔ Caspase 1 ↓ Cleaved caspase 1 ↓ GSDMD‐NT	↓ IL‐1β ↓ IL‐18	↓ LncRNA Neat1		
NRCMs cells	H/R (H: 6 h, R: 4 h)	Cotreatment with Carbachol 0.5 × 10 M at the onset of H/R (compared with control)	N/A	↓ NLRP3 ↓ Cleaved caspase 1/Caspase 1 ↓ GSDMD‐NT/GSDMD‐NT	N/A	↓ OGDHL ↓ ATP production ↓ mtROS	Carbachol alleviated NLRP3‐mediated pyroptosis through the M2AChR/OGDHL/ROS axis in H/R cardiomyocyte.	[87]
		Cotreatment with Carbachol 0.5 × 10 M + Otenzepad 0.5 × 10 M at the onset of H/R (compared with control)	N/A	N/A	N/A	↑ OGDHL ↑ ATP production		
		Cotreatment with Carbachol 0.5 × 10 M + OGDHL overexpression (compared with control)	N/A	↑ NLRP3 ↑ Cleaved caspase 1/Caspase 1 ↑ GSDMD‐NT/GSDMD‐NT	N/A	↑ OGDHL ↑ ATP production ↑ mtROS		
HL‐1 cells	H/R (H: 8 h, R: 24 h)	Pretreatment with Epigallocatechin gallate 40 μM 12 h prior to H/R (compared with control)	↑ Cell viability ↓ LDH cytotoxicity	↓ AIM2 ↓ ASC ↓ Cleaved caspase 1 ↓ GSDMD‐NT	↓ IL‐1β ↓ IL‐18	↓ Lnc RNA MEG3 ↔ TAF15	Epigallocatechin gallate‐mitigated cardiomyocytes pyroptosis by inhibiting LncRNA MEG3/TAF15/AIM2 axis in H/R cardiomyocyte.	[60]
NMCMs cells	H/R (H: 24 h, R: 12 h)	Cotreatment with exo‐miR‐182‐5p mimic (dose no shown) (compared with control)	↑ Cell viability ↑ Cell survival	↓ NLRP3 ↓ ASC ↓ Caspase 1 ↓ Cleaved caspase 1 ↓ GSDMD ↓ GSDMD‐NT	↓ IL‐1β ↓ IL‐18	↓ LDH ↓ ROS	Exosomal miR‐182‐5p inhibited inflammation and pyroptosis activation through the inhibition of GSDMD in H/R cardiomyocyte.	[45]
		Cotreatment with Exo‐miR‐182‐5p mimic + Exo‐miR‐182‐5p mimic inhibitor (dose no shown) (compared with control)	↓ Cell viability ↓ Cell survival	↑ NLRP3 ↑ ASC ↑ Caspase 1 ↑ Cleaved caspase 1 ↑ GSDMD ↑ GSDMD‐NT	↑ IL‐1β ↑ IL‐18	↑ LDH ↑ ROS		
		Cotreatment with Exo‐miR‐182‐5p mimic inhibitor + GSDMD knock down (dose no shown) (compared with shGSDMD)	↓ Cell viability ↓ Cell survival	↑ NLRP3 ↑ ASC ↑ Caspase 1 ↑ Cleaved caspase 1 ↑ GSDMD ↑ GSDMD‐NT	↑ IL‐1β ↑ IL‐18	↑ LDH ↑ ROS		
H9c2 cells	Hypoxia 2 h	Cotreatment with Melatonin 10 μM in hypoxic environment (compared with control)	↓ LDH cytotoxicity	↓ NLRP3 ↓ Caspase 1 ↓ Cleaved caspase 1 ↓ GSDMD‐NT	↓ IL‐1β ↓ IL‐18	↓ TLR4 ↓ p65	Melatonin protect against NLRP3 inflammasome‐mediated pyroptosis through the inhibition of the TLR4/NF‐κB/p65 signalling pathway in hypoxic cardiomyocyte.	[75]
H9c2 cells	Hypoxia + TNF‐α 40 ng/mL 12 h	Cotreatment with Liraglutide 100 nM in hypoxic environment (compared with control)	↑ Cell viability ↓ LDH cytotoxicity	↓ NLRP3 ↓ Caspase 1 ↓ Cleaved caspase 1 ↓ GSDMD‐NT	N/A	↑ SIRT1 ↓ NOX4	Liraglutide attenuated NLRP3‐mediated pyroptosis through SIRT1/NOX4/ROS signalling pathway in cardiomyocyte in hypoxic condition.	[77]
H9c2 cells	Hypoxia + TNF‐α 40 ng/mL 12 h	Pretreatment with Rosuvastatin 20 μM 2 h prior to hypoxia (compared with control)	↑ Cell viability	↓ NLRP3 ↓ Cleaved caspase 1 ↓ GSDMD‐NT	N/A	↓ LDH ↓ PI‐positive nuclei ↓ mitoSOX	Rosuvastatin protected against NLRP3‐mediated pyroptosis by regulating mitochondrial ROS in hypoxic cardiomyocyte.	[76]
		Pretreatment with VX‐765 20 μM 2 h prior to hypoxia (compared with control)	↑ Cell viability	N/A	N/A	↓ LDH ↓ PI‐positive nuclei		
		Pretreatment with *N*‐acetylcysteine 5 mM 2 h prior to hypoxia (compared with control)	↑ Cell viability	↓ NLRP3 ↓ Cleaved caspase 1 ↓ GSDMD‐NT	N/A	↓ LDH ↓ PI‐positive nuclei		
NRVMs cells	Hypoxia 12 h	Co‐treatment with hydrogen culture medium in hypoxic environment (compared with control)	↑ Cell viability ↓ LDH cytotoxicity	↓ NLRP3 ↓ ASC ↓ Cleaved caspase 1 ↓ GSDMD	↓ IL‐1β	N/A	Both H_2_ and NLRP3‐inhibitor MCC950 treatment alleviated the hypoxia‐induced NPRP3‐mediated pyroptosis and the combine treatment did not provide the additional protective effect.	[79]
		Cotreatment with MCC950 10 μM in hypoxic environment (compared with control)	↑ Cell viability ↓ LDH cytotoxicity	↓ NLRP3 ↓ ASC ↓ Cleaved caspase 1 ↓ GSDMD	↓ IL‐1β	N/A		
		Cotreatment with hydrogen culture medium + MCC950 10 μM in hypoxic environment (compared with control)	↑ Cell viability ↓ LDH cytotoxicity	↓ NLRP3 ↓ ASC ↓ Cleaved caspase 1 ↓ GSDMD	↓ IL‐1β	N/A		
NMCMs cells	Hypoxia 24 h	Cotreatment with Kanglexin 10 μM in hypoxic environment (compared with control)	N/A	↓ NLRP3 ↓ Cleaved caspase 1 ↓ GSDMD ↓ GSDMD‐NT	↓ IL‐1β ↓ IL‐18	↓ TUNEL‐positive nuclei ↓ PI positive nuclei	Kanglexin protected against NLRP3‐mediated pyroptosis and apoptosis in hypoxic condition.	[80]
H9c2 cells	OGD 12 h	Co‐treatment with Ginsenoside Rh2 2 μM in OGD condition (compared with control)	↑ Cell viability	↓ NLRP3 ↓ Cleaved caspase 1 ↓ GSDMD‐NT	↓ IL‐1β ↓ IL‐18	↓ HMGB1	Ginsenoside Rh2 decreased HMGB1 protein expression, restrained the occurrence of the inflammatory cascade thus preventing the pyroptosis activation in the cardiomyocyte under OGD condition.	[81]
H9c2 cells	OGD 36 h	Co‐treatment with *N*‐acetyl‐cysteine 50 μM in OGD condition (compared with control)	N/A	↓ NLRP3 ↓ ASC ↓ Caspase1 ↓ Cleaved caspase 1 ↓ GSDMD ↓ GSDMD‐NT		↓ LDH ↓ ROS ↓ p‐NF‐kB p65 ↑ SOD	*N*‐acetyl‐cysteine reduced oxidative stress thus mitigated p‐NF‐kB/NLRP3/GSDMD‐mediated pyroptosis in the cardiomyocyte under OGD condition.	[89]
		Co‐treatment with Pyrrolidine dithiocarbamate 25 μM in OGD condition (compared with control)	N/A	↓ NLRP3 ↓ ASC ↓ Caspase1 ↓ Cleaved caspase 1 ↓ GSDMD ↓ GSDMD‐NT		↔ p‐NF‐kB p65		

## 8 Modulating Canonical Pyroptosis as a Potential Therapeutic Target in IHD: Reports Across In Vivo Studies

Given that pyroptosis is closely associated with robust inflammation, and inflammation induces and exacerbates pyroptosis, numerous studies have investigated the potential repurposing of routinely used anti‐inflammatory agents for targeting pyroptosis‐mediated IHD [25, 37]. Dong et al. [85] demonstrated that aspirin, a widely used nonsteroidal anti‐inflammatory drug (NSAID), effectively mitigated cardiac dysfunction by suppressing inflammation and pyroptosis, thereby improving cardiac function in cardiac I/R rats. They also reported that the combination of aspirin with gastrodin, a polyphenol extract known for its antioxidant and anti‐inflammatory properties, more effectively suppressed inflammation and pyroptosis than either given as a monotherapy, thus improving cardiac function in I/R rats [85]. Colchicine, one of the first‐line medications for acute gout and Behçet's disease, was indicated as a potential therapy for reducing significant cardiovascular events after MI, as demonstrated in the COLCOT trial [112, 113]. Mechanistically, colchicine inhibited the intracellular assembly and activation of the NLRP3 inflammasome, ultimately leading to a reduction in inflammation [114]. Wang et al. [86] reported that intravenous administration with colchicine‐containing nanoparticles after ischaemia significantly reduced inflammation and GSDMD‐mediated pyroptosis in I/R rats.

Another study reported that pretreatment with nicorandil, a coronary vasodilatory drug, demonstrated cardioprotective effects evidenced by enhancement in cardiac function, and a reduction in infarct size and inflammation [23]. Mechanistically, nicorandil significantly inhibited TLR4 expression, thus consequently mitigating the TLR4/MyD88/NF‐κB/NLRP3 signalling pathway and ultimately alleviating myocardial pyroptosis in AMI rats [23]. Interestingly, it had been reported that molecular hydrogen therapy (H_2_) demonstrated very promising efficacy as an antioxidant and anti‐inflammatory treatment against several oxidative stress, inflammatory and ageing‐related pathologies [115, 116]. In the heart, H_2_ inhalant could attenuate cardiac dysfunctions in AMI rats by regulating oxidative stress, NLRP3‐dependent pyroptosis and inflammation [88]. Other pharmacological agents and natural compounds such as insulin, chlorogenic acid, dexmedetomidine, emodin, gastrodin, geniposide, ginsenoside Rh2, epigallocatechin gallate, astragaloside‐IV and tanshinone IIA provide an antipyroptotic effect in animal models of IHD mainly through the inhibition of the NLRP3 inflammasomes [47, 60, 68, 72, 84, 91, 117, 118, 119]. These findings underscored and repurposed the potential efficacy of routine medications and natural‐based compounds against pyroptosis in the context of IHD.

Neurohormonal disturbance is known to cause sympathetic overactivation and has been associated with adverse prognosis in cardiovascular diseases and IHDs [120]. During cardiac pathologies (e.g., acute and chronic ischaemia, I/R injury and cardiomyopathies), the renin–angiotensin–aldosterone system (RAAS) is frequently hyperactivated as a compensatory mechanism to increase fluid retention [45, 46]. However, excessive RAAS activation significantly compromises cardiac function, ultimately leading to heart failure [45, 46]. Moreover, excessive activation of angiotensin receptors has been reported to induce intracellular oxidative stress and calcium overload, leading to myocardial cell death through both canonical and noncanonical pyroptosis pathways [47, 48, 49, 121, 122, 123] . In several cardiac pathologies, angiotensin ІІ is acknowledged as being a significant contributor to the pathogenesis of cardiac inflammation, adverse remodelling and heart failure [124, 125]. Despite recent medical advances, the administration of angiotensin converting enzyme (ACE) inhibitors and angiotensin II receptor blockers (ARBs) has demonstrated considerable effectiveness in decreasing mortality and morbidity, while also enhancing clinical outcomes among individuals with heart failure [126, 127]. Supporting these findings, posttreatment with an ACE inhibitor captopril could inhibit myocardial pyroptosis via the TLR4/NF‐κB p65/NLRP3 pathway, thus alleviating cardiac dysfunction and remodelling in post‐MI rats [117]. Likewise, sacubitril/valsartan (LCZ696), an angiotensin receptor neprilysin inhibitor (ARNI), demonstrated notable suppression of NLRP3‐mediated pyroptosis, leading to reduced infarction and improved cardiac function in AMI rats [90]. Interestingly, Lu et al. [87] reported that stimulation of muscarinic acetylcholine receptor M2 (M2AChR) using magnetic vagus nerve stimulation could alleviate the accumulation of mtROS by inhibiting mitochondrial multienzyme 2‐oxo‐glutarate dehydrogenase (OGDH), thereby mitigating NLRP3‐mediated pyroptosis in I/R rats. Therefore, despite the detrimental effects of sympathetic overactivation and the significant role of angiotensin II in IHD pathogenesis advancements in pharmacotherapy, such as ACE inhibitors, ARBs and novel interventions targeting the muscarinic receptors, offer promising avenues for mitigating myocardial pyroptosis and improving functional outcomes in IHD‐related conditions.

**FIGURE 3:**
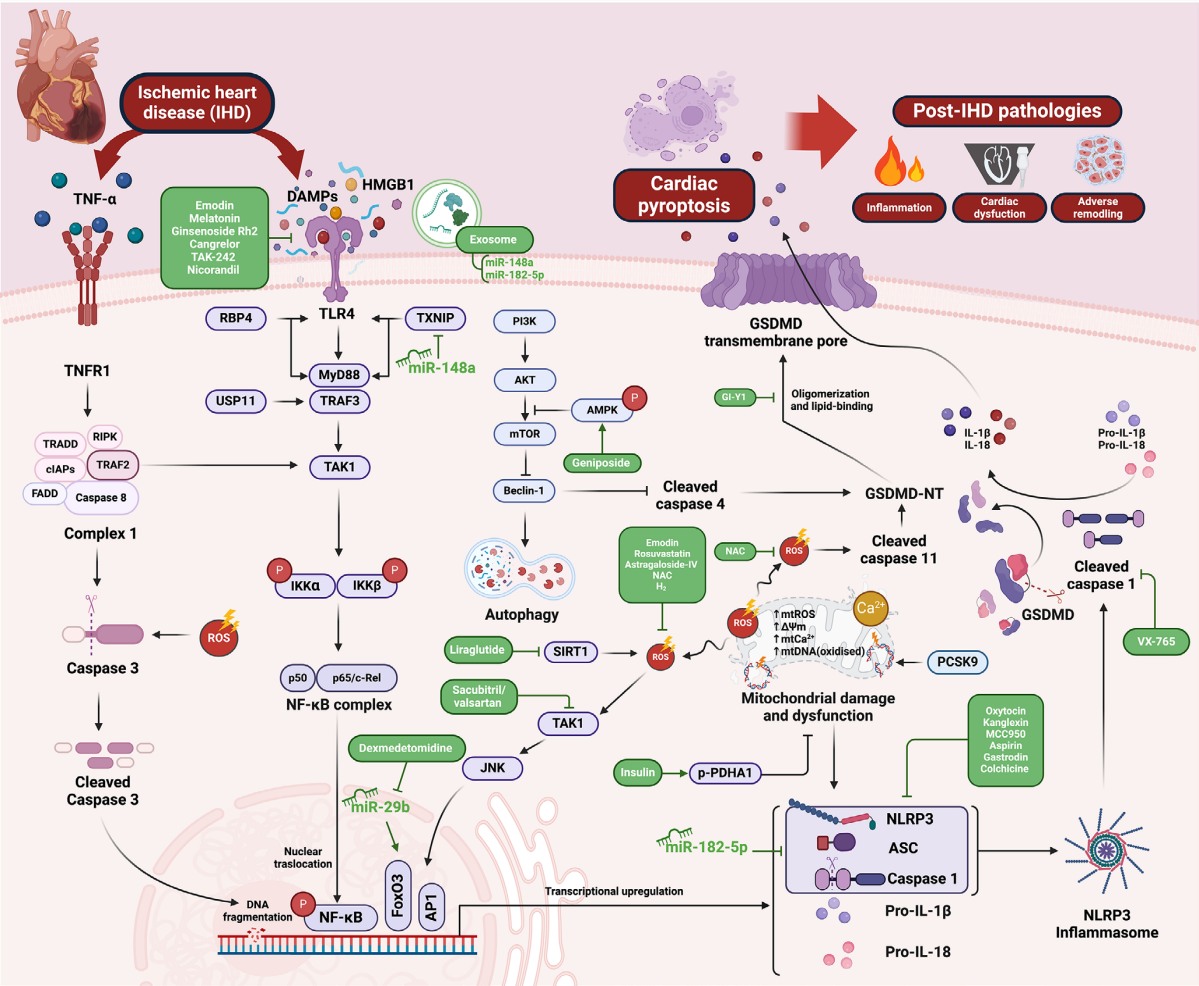
Potential therapeutic targets for pyroptosis in IHD. IHD causes canonical pyroptosis through the binding of DAMPs (e.g., HMGB1) to their corresponding PRRs (e.g., TLR4). Thereby, the activation of TLR4/MyD88/nF‐κB/NLRP3 leads to the cleavage of caspase 1 and GSDMD transmembrane localisation. The activation of pyroptosis causes subsequent cell lysis, robust inflammation, cardiac dysfunction, adverse remodelling and subsequent heart failure. Intrinsic stimuli include ROS, mitochodrial damage and dysfunction and intracellular Ca^2+^‐augmented pyroptosis through the activation of the NLRP3 inflammasome. The impairment of the autophagy flux process also caused pyroptosis through the overactivation of caspase 4. Emodin, melatonin, gindenoside Rh2, Cangrelor, TAK‐242 and Nicorandil mitigated cardiac pyroptosis by inhibiting TLR4 activation. Emodin, rosuvastatin, astragaloside‐IV, NAC and H_2_ conferred antioxidative properties and thus prevented ROS/NF‐κB/NLRP3/caspase 1‐mediated pyroptosis. Oxytocin, kanglexin, MCC950, aspirin, gastrodin and cochicine mitigated NLRP3 inflammasome activation. VX‐765 inhibited the catalytic activity of caspase 1. GI‐Y1 inhibited GSDMD‐NT oligomerisation and lipid membrane binding. ASC, apoptosis‐associated speck‐like protein containing a CARD; DAMPs, damage‐associated molecular patterns; ECM, extracellular matrix; GSDMD, gasdermin D; GSDMD‐NT, *N*‐terminal domain of gasdermin D; HMGB1, high mobility group box 1; IHD, ischaemic heart disease; IKKα/β, IKKalpha kinase α/β; IL‐18, interleukin 18; IL‐1β, interleukin 1β; mtDNA, mitochondrial DNA; mtROS, mitochondrial ROS; Myd88, myeloid differentiation factor 88; NAC, *N*‐acetylcysteine; NF‐κB, nuclear factor kappa B; NLRP3, nucleotide‐binding domain, leucine‐rich‐containing family, pyrin domain‐containing‐3; PRRs, pathogen recognition receptors; TAK1, transforming growth factor‐β activated kinase 1; TLR4, toll‐like receptor 4; TNFR, tumour necrosis factor receptor‐associated factor; TNF‐α, tumour necrosis factor alpha.

The application of specific inhibitors targeting the NLRP3/caspase 1/GSDMD axis has shown promising cardioprotection against pyroptosis in in vitro studies. In line with the in vitro findings, pretreatment with MCC950 showed a level of protection against cardiac injuries, fibrosis and contractile dysfunction in CME‐induced AMI mice [76]. Administration of VX‐765 immediately postischaemia and prior to reperfusion significantly resulted in the preservation of cardiac function, reduced infarction size and mitigation of inflammation in I/R rats [14, 19]. Additionally, treatment with GI‐Y1 effectively attenuated GSDMD‐mediated myocardial pyroptosis and reduced infarct size in I/R mice [34]. The detailed mechanisms are comprehensively outlined in Table 5. Collectively, the application of novel targeted inhibition of the NLRP3/caspase 1/GSDMD axis holds significant promise as a therapeutic strategy for mitigating myocardial injury and inflammation in IHD, as evidenced by the robust protective effects observed in both in vitro and in vivo models. These findings underscore the potential of these inhibitors as valuable tools in the treatment of IHD. A summary of the potential therapeutic targeting of pyroptosis in IHD is illustrated in Figure 3.

**TABLE 5:** Evidence of interventions on pyroptosis in IHD models: reports across ex vivo and in vivo studies.

Study models	Methods	Intervention	Major findings				Interpretation	Reference
			Functional outcome	Pyroptosis	Inflammation	Other biochemical markers		
Langendorff isolated rat heart perfusion	Cardiac I/R (I: 30 min, R: 1 h)	Cotreatment with Insulin 0.5 IU/L IV along the reperfusion (compared with control)	↑ LVEDP ↑ dP/dt(max) ↓ dP/dt(min)	↓ Cleaved caspase 1 ↓ GSDMD‐NT	↓ IL‐1β ↓ IL‐18	↓ %Infarction ↓ TUNEL‐positive nuclei ↓ LDH ↓ CK ↑ PDH ↑ ATP ↓ Lactate ↓ p‐PDHA1 (Ser293).	Insulin improved myocardial metabolism via upregulation of p‐PDHA1 and also alleviated pyroptosis‐induced inflammation during cardiac I/R. PDHA1 knockdown enhanced NLRP3 expression and abrogated antipyroptotic effect of insulin during cardiac I/R.	[118]
		Pretreatment with PDHA1 knock down + Cotreatment with Insulin 0.5 IU/L IV along the reperfusion (compared with WT control)	↓ LVEDP ↓ dP/dt(max) ↑ dP/dt(min)	↑ NLRP3 ↑ Cleaved caspase 1 ↑ GSDMD‐NT	↑ IL‐1β ↑ IL‐18	↑ TUNEL‐positive nuclei ↑ LDH ↑ CK		
SD rats	Coronary microembolisation by polyethylene microspheres intraventricular injection for 6 h	Pretreatment with TAK‐242 2 mg/kg IV 30 min prior to CME (compared with control)	↑ %LVEF, %LVFS ↑ CO ↓ LVEDd	↓ NLRP3 ↓ ASC ↓ Caspase 1	↓ IL‐1β ↓ IL‐18 ↓ TNF‐α	↓ %Micro infarction ↓ Serum cTnI ↓ TLR4 ↓ MyD88 ↓ NF‐κB p65	Pretreatment with TLR4 inhibitor TAK‐242 mitigated myocardial injuries and inflammation thought the inhibition of TLR4/Myd88/NF‐κB signalling pathway in CME rat.	[83]
C57BL/6 mice	Coronary microembolisation by polyethylene microspheres intraventricular injection for 72 h	Pretreatment with Rosuvastatin 40 mg/kg/day 3 days prior and 3 days after CME (compared with control)	↑ %LVEF, %LVFS	↓ NLRP3 ↓ Cleaved caspase 1 ↓ GSDMD‐NT	↓ IL‐1β	↓ %Micro infarction ↓ LDH ↓ Collagen content ↓ SDHA, SDHB ↓ DHE ↓ MitoSOX	Rosuvastatin given before and after CME alleviated mitochondria damage, mt ROS, NLRP3‐mediated myocardial pyroptosis thus prevented cardiac inflammation, and cardiac dysfunction in AMI mice.	[76]
C57BL/6 mice	Coronary microembolisation by polyethylene microspheres intraventricular injection for 72 h	Pretreatment with MCC950 20 mg/kg/day 3 days prior and 3 days after CME (compared with control)	↑ %LVEF, %LVFS	N/A	N/A	↓ %Micro infarction ↓ LDH ↓ Collagen content	Pretreatment with NLRP3 inhibitor MCC950 protected against CME‐induced cardiac injuries, cardiac fibrosis, and contractile dysfunction in AMI mice.	[76]
C57BL/6 mice	Cardiac I/R (I: 30 min, R: 1 h)	Pretreatment with Chlorogenic acid 30 mg/kg/day 4 weeks prior to I/R (compared with control)	N/A	↓ NLRP3 ↓ ASC ↔ Caspase 1 ↓ Cleaved caspase 1 ↔ GSDMD ↓ GSDMD‐NT	↓ IL‐1β ↓ IL‐18	↓ %Infarction ↓ LDH ↓ cTnT ↓ CK‐MB ↓ LncRNA Neat1	Chlorogenic acid inhibited Lnc Neat1/NLRP3 inflammasome‐mediated pyroptosis in I/R mice.	[72]
SD rats	Cardiac I/R (I: 30 min, R: 2 h)	Pretreatment with Dexmedetomidine 2 μg/kg IP 30 min prior to I/R (compared with control)	N/A	↓ NLRP3 ↓ ASC ↓ Cleaved caspase 1	↓ IL‐1β ↓ IL‐18	↓ %Infarction ↓ cTnI ↓ CK‐MB ↓ TUNEL positive nuclei ↓ miR‐29b ↓ FoxO3a ↓ ARC	Dexmedetomidine ameliorated myocardial injuries and pyroptosis activation via suppressing miR‐29b‐activated FoxO3a/ARC axis.	[84]
		Pretreatment with Dexmedetomidine 2 μg/kg IP 30 min + miR‐29b agomir (10 nM) IV 3 days prior to I/R (compared with WT control)	N/A	↑ NLRP3 ↑ ASC ↑ Cleaved caspase 1	↑ IL‐1β ↑ IL‐18	↑ %Infarction ↑ cTnI ↑ CK‐MB ↑ TUNEL positive nuclei		
		Pretreatment with Dexmedetomidine 2 μg/kg IP 30 min + miR‐29b antagomir (10 nM) IV 3 days prior to I/R (compared with WT control)	N/A	↓ NLRP3 ↓ ASC ↓ Cleaved caspase 1	↓ IL‐1β ↓ IL‐18	↓ %Infarction ↓ cTnI ↓ CK‐MB ↓ TUNEL positive nuclei		
SD rats	Cardiac I/R (I: 30 min, R: 2 h)	Pretreatment with Emodin 20 mg/kg IP 1 h prior to I/R (compared with control)	N/A	↔ GSDMD ↓ GSDMD‐NT	↓ IL‐1β	↓ IS/AAR ↔ AAR/LV ↓ Disorders and swollen cells	Emodin pretreatment ameliorated GSDMD‐mediated myocardial pyroptosis and reduced infarction in I/R rats.	[68]
Kunming mice	Cardiac I/R (I: 30 min, R: 2 h)	Pretreatment with Potassium oxonate in 0.5% sodium carboxymethyl cellulose 300 mg/kg IP for 14 prior to I/R (compared with control)	N/A	↑ NLRP3 ↑ ASC ↑ Caspase1 ↑ GSDMD	↑ IL‐1β	↑ %Infarction ↑ Serum LDH ↑ Serum CK‐MB ↑ Serum uric acid ↑ TUNEL positive nuclei ↑ Caspase 3	Uric acid aggravates myocardial pyroptosis in I/R rats.	[111]
C57BL/6 mice	Cardiac I/R (I: 30 min, R: 3 h)	Posttreatment with exo‐miR‐182‐5p mimic 10 μg intracardiac injection after LAD ligation (compared with WT control)	↑ %LVEF, %LVFS ↓ LVEDd, LVESd	↓ NLRP3 ↓ ASC ↓ Caspase 1 ↓ Cleaved caspase 1 ↓ GSDMD ↓ GSDMD‐NT	↓ IL‐1β ↓ IL‐18	↓ %Infarction ↓ LDH ↓ ROS ↑ miRNA‐182‐5p	Exosomal miR‐182‐5p mitigated I/R‐induced myocardial pyroptosis and cardiac dysfunction in I/R mice.	[45]
C57BL/6 mice	Cardiac I/R (I: 30 min, R: 4 h)	Pretreatment with GI‐Y1 50 mg/kg IP 2 h prior to I/R (compared with control)	N/A	↓ GSDMD‐NT	↓ IL‐1β ↓ IL‐18	↓ IS/AAR ↔ AAR/LV ↓ LDH ↓ CK‐MB	Administration with GI‐Y1 prior or after I/R mitigated GSDMD‐mediated myocardial pyroptosis and infarct size in I/R mice.	[34]
	Cardiac I/R (I: 30 min, R: 4 h)	Posttreatment with GI‐Y1 50 mg/kg IP 1 min after onset of reperfusion (compared with control)	↑ %LVEF, %LVFS	↓ GSDMD‐NT	↓ IL‐1β ↓ IL‐18	↓ IS/AAR ↔ AAR/LV		
		Posttreatment with GI‐Y1 50 mg/kg IP 1 min after onset of reperfusion + GSDMD knockout (compared with control)	N/A	↔ GSDMD‐NT	↔ IL‐1β ↔ IL‐18	↔ IS/AAR ↔ AAR/LV		
	Cardiac I/R (I: 30 min, R: 2 week)	Posttreatment with GI‐Y1 50 mg/kg IP 1 min after onset of reperfusion (compared with control)	↑ %LVEF, %LVFS	↓ GSDMD‐NT	↓ IL‐1β ↓ IL‐18	↓ IS/AAR ↔ AAR/LV		
SD rats	Cardiac I/R (I: 45 min, R: 2 h)	Pretreatment with Aspirin 50 mg/kg/day IG 3 days prior to I/R (compared with control)	↑ LVEDP ↑ dP/dt(max) ↓ dP/dt(min)	↓ NLRP3 ↓ ASC ↓ Caspase 1 ↓ GSDMD	↓ COX I ↓ COX II ↓ TNF‐α ↓ IL‐6	↓ %Infarction ↓ cTnI	Aspirin alleviates cardiac dysfunction by suppressing inflammation and pyroptosis thus improved cardiac function in cardiac I/R rats.	[85]
SD rats	Cardiac I/R (I: 45 min, R: 2 h)	Aspirin 50 mg/kg/day IG + Gastrodin 200 mg/kg IP 3 days prior to ischaemia (compared with control)	↑↑ LVEDP ↑↑ dP/dt(max) ↓↓ dP/dt(min)	↓↓ NLRP3 ↓↓ ASC ↓↓ Caspase 1 ↓↓ GSDMD	↓↓ TNF‐α ↓↓ IL‐6	↓↓ %Infarction ↓↓ cTnI	Combined treatment with aspirin and gastrodin mitigates cardiac dysfunction by suppressing inflammation and pyroptosis in cardiac I/R rats.	[85]
C57BL/6J mice	Cardiac I/R (I: 45 min, R: 4 h)	Pretreatment with Geniposide 10 or 20 mg/ kg IP 30 mins prior to ischaemia (compared with control)	↓ %LVEF, %LVFS (dose dependently) ↑ LVEDd, LVESd (dose dependently)	↓ NLRP3 ↓ ASC (dose dependently) ↓ Cleaved caspase 1 ↓ Caspase 1 activity (dose dependently) ↓ GSDMD (dose dependently)	↓ IL‐1β (dose dependently) ↓ IL‐18 (dose dependently)	↓ %Infarction (dose dependently) ↓ TUNEL positive nuclei ↓ LDH (dose dependently) ↓ CK (dose dependently) ↓ CK‐MB (dose dependently) ↑ p‐AMPK/AMPK (dose dependently) ↑ p‐ACC/ACC (dose dependently) ↓ ROS (dose dependently) ↑ SOD	Geniposide suppresses myocardial apoptosis and NLRP3 inflammasome‐mediated pyroptosis via the activation of AMPK signalling pathway in a dose dependent fashion in cardiac I/R mice.	[47]
SD rats	Cardiac I/R (I: 60 mins, R: 30 mins)	Pretreatment with VX‐765 32 mg/kg IV 5 mins prior to reperfusion (compared with control)	↑ %LVFS	N/A	↓ Cleaved IL‐1β	↓ %Infarction ↓ Aldolase‐A ↓ Hexokinase II ↓ Complex I activity ↑ Complex III activity	Caspase 1 inhibitor VX‐765 immediately before reperfusion preserved cardiac function, reduced infarction and inflammation in I/R rats.	[14]
SD rats	Cardiac I/R (I: 60 min, R: 30 min)	Pretreatment with Cangrelor 60 μg/kg IV 10 min prior to reperfusion (compared with control)	N/A	N/A	N/A	↓ %Infarction	Caspase 1 inhibitor VX‐765 in combination with P2Y_12_ receptor antagonist Cangrelor given immediately before reperfusion resulted in marked preservation of left ventricular function and reduced infarct size in I/R rats.	[14]
		Pretreatment with Ticagrelor 30 μg/kg IP 10 min prior to reperfusion (compared with control)	N/A	N/A	N/A	↓ %Infarction		
		Ischaemic preconditioning 3 cycles of 30 s of reperfusion followed by 30 s of reocclusion	N/A	N/A	N/A	↓ %Infarction		
		Pretreatment with VX‐765 32 mg/kg IV and Cangrelor 60 μg/kg IV 10 min prior to reperfusion (compared with control)	N/A	N/A	N/A	↓↓ %Infarction		
		Pretreatment with VX‐765 32 mg/kg IV and Ticagrelor 30 μg/kg IP 10 min prior to reperfusion (compared with control)	N/A	N/A	N/A	↓↓ %Infarction		
		Ischaemic preconditioning 3 cycles of 30 s of reperfusion followed by 30 s of reocclusion and cangrelor 60 μg/kg IV 10 mins prior to reperfusion (compared with control)	N/A	N/A	N/A	↓ %Infarction		
SD rats	Cardiac I/R (I: 30 min, R: 2 h)	Co‐treatment with Magnetic vagus nerve stimulation 100 mT, 20 Hz along the reperfusion period (compared with control)	↑ %LVEF, %LVFS	↓ NLRP3 ↓ Cleaved caspase1/Caspase 1 ↓ GSDMD‐NT/GSDMD	↓ IL‐1β ↓ IL‐18 ↓ IL‐6 ↓ TNF‐α	↓ IS/AAR ↓ cTnT ↓ LDH ↓ CK‐MB ↓ TUNEL positive nuclei ↓ Incidence of VF ↓ Duration of VF ↓ OGDHL ↓ ROS	Magnetic vagus nerve stimulation alleviates cardiac I/R injuries by the inhibiting NLRP3‐mediated pyroptosis through the M2AChR/OGDHL/ROS axis.	[87]
		Co‐treatment with Magnetic vagus nerve stimulation 100 mT, 20 Hz along the reperfusion period + OGDHL knockdown (compared with wildtype)	↓ %LVEF, %LVFS	↑ NLRP3 ↑ Cleaved caspase 1/Caspase 1 ↑ GSDMD‐NT/GSDMD	↑ IL‐1β ↑ IL‐18 ↑ IL‐6 ↑ TNF‐α	↑ IS/AAR ↑ cTnT ↑ LDH ↑ CK‐MB ↑ TUNEL positive nuclei ↑ Incidence of VF ↑ Duration of VF ↑ OGDHL ↑ ROS		
		Cotreatment with Magnetic vagus nerve stimulation 100 mT, 20 Hz along the reperfusion period + Otenzepad (compared with control)	↓ %LVEF, %LVFS	↑ NLRP3 ↑ Cleaved caspase 1/Caspase 1 ↑ GSDMD‐NT/GSDMD	↑ IL‐1β ↑ IL‐18 ↑ IL‐6 ↑ TNF‐α	↑ IS/AAR ↑ TUNEL positive nuclei ↑ OGDHL ↑ ROS		
SD rats	Cardiac I/R (I: 30 min, R: 24 h)	Pretreatment with M2‐Exosome 2–3 μg/rat IV 2 h prior to I/R (compared with control)	N/A	↓ NLRP3 ↓ ASC	N/A	↓ IS/AAR ↔ AAR/LV ↓ Disorders and swollen cells ↓ Serum LDH ↓ Serum CK ↓ Serum CK‐MB ↓ Intracellular Ca^2+^ ↓ IP3R ↓ SERCA2a ↓ TXNIP ↓ TLR4 ↓ MyD88 ↓ p‐NF‐κB p65/ NF‐κB p65 ↓ p‐lκBα/lκBα ↑ miR‐18a	M2‐Exosome promoted miR‐148a expression thus ameliorated myocardial pyroptosis, cardiac injuries, myocardial Ca^2+^ overload and infarction through TXNIP/TLR4/MyD88/NF‐κB/NLRP3 signalling pathway in I/R rats.	[110]
Wistar rats	Cardiac I/R (I: 45 min, R: 7 days)	Posttreatment with Colchicine‐containing Nanoparticles 2 mg/kg IV 30 min, 48 h, or 96 h after LAD ligation (compared with control)	N/A	↓ Cleaved caspase 1 ↓ GSDMD ↓ GSDM‐NI	↓ IL‐1β ↓ Cleaved IL‐1β ↓ TNF‐α ↓ CRP ↑ IL‐10	↓ %Infarction	Colchicine‐containing nanoparticles protected against cardiac I/R injury by reducing inflammation and pyroptosis in rats.	[86]
Wistar rats	Permanent LAD ligation for 24 h	Posttreatment with 2% H_2_ inhalant for 24 h after LAD ligation (compared with control)	↑ %LVEF, %LVFS ↓ LVIDd, LVIDs	↓ NLRP3 ↓ ASC ↓ Cleaved caspase 1 ↓ GSDMD	↓ IL‐1β ↓ Inflammatory cells infiltration	↓ %Infarction ↓ Serum cTnI, BNP ↓ 8‐OHdG‐positive cells ↓ ROS ↓ MDA ↓ TUNEL‐positive nuclei	[Post‐treatment with H_2_ inhalant improved cardiac functions, reduced oxidative stress, cardiac injuries, apoptosis, and pyroptosis in I/R rats.	[88]
SD rats	Permanent LAD ligation for 72 h	Pretreatment with Nicorandil 15 mg/kg/d IG for 7 days prior to LAD ligation (compared with control)	↑ %LVEF, %LVFS ↓ LVEDd, LVESd	↓ NLRP3 ↓ ASC ↓ Cleaved caspase 1 ↔ GSDMD ↓ GSDMD‐NT	↓ IL‐1β ↓ IL‐18	↓ %Infarction ↓ Serum cTnI, LDH ↑ Myocardial ATP, ADP, AMP ↓ TLR4 ↓ MyD88 ↓ NF‐κB p65	Pretreatment with Nicorandil improved cardiac functions, infarction, and inflammation by inhibiting TLR4/MyD88/NF‐κB/NLRP3 signalling pathway thus reduced myocardial pyroptosis in rats with AMI. Combined treatment of Nicorandil and TLR4 inhibitor TAK242 did not provide the better cardioprotection of Nicorandil monotherapy in AMI rats.	[23]
		Pretreatment with Nicorandil 15 mg/kg/d IG for 7 days + TAK242 2 mg/kg IV 30 min prior to LAD ligation (compared with control)	↑ %LVEF, %LVFS ↓ LVEDd, LVESd	↓ NLRP3 ↓ ASC ↓ Cleaved caspase 1 ↔ GSDMD ↓ GSDMD‐NT	↓ IL‐1β ↓ IL‐18	↓ %Infarction ↓ Serum cTnI, LDH ↓ TLR4 ↓ MyD88 ↓ NF‐κB p65		
C57BL/6 mice	Permanent LAD ligation for 72 h	Posttreatment with VX765 50 mg/kg IP 10 min after LAD ligation (compared with control)	↑ %LVEF	↓ Caspase1 ↓ Cleaved caspase 1	N/A	↓ %Infarction	Caspase 1 inhibitor VX‐765 treatment in the extremely early stage (10 min) of AMI significantly reduces the infarction region in AMI mice.	[19]
SD rats	Permanent LAD ligation 1 week	Posttreatment with Sacubitril/valsartan (LCZ696) 68 mg/kg PO 10 for 7 days LAD ligation (compared with control)	↑ %LVEF, %LVFS ↓ LVIDd, LVIDs	↓ NLRP3 ↓ Caspase1 ↓ Cleaved caspase 1 ↓ GSDMD	↓ IL‐1β ↓ IL‐18	↓ %Infarction ↓ %Collagen volume fraction	Sacubitril/valsartan (LCZ696) suppressed NLRP3‐mediated pyroptosis and the expression TAK1/JNK pathway thus improved cardiac function in AMI rats.	[90]
SD rats	Permanent LAD ligation 2 weeks	Posttreatment with Ginsenoside Rh2 2 mg/kg/day IP for 2 weeks (compared with Sham)	↓↓↓ HR ↓↓↓ SBP, DBP, MAP ↓↓↓ LVESP, LVEDP	↑↑↑ NLRP3 ↑↑↑ Caspase 1 ↑↑↑ GSDMD	↑ IL‐1β	↑↑↑ LDH ↑↑↑ CK ↑↑↑ CK‐MB	Ginsenoside Rh2 dose dependently mitigated cardiac pyroptosis, reduced myocardial injuries thus improved haemodynamics and cardiac function in AMI rats.	[119]
SD rats	Permanent LAD ligation 2 weeks	Posttreatment with Ginsenoside Rh2 4 mg/kg/day IP for 2 weeks (compared with Sham)	↓↓ HR ↓↓ SBP, DBP, MAP ↓↓ LVESP, LVEDP	↑↑ NLRP3 ↑↑ Caspase 1 ↑↑ GSDMD	↑↑↑ IL‐1β	↑↑ LDH ↑↑ CK ↑↑ CK‐MB	Ginsenoside Rh2 dose dependently mitigated cardiac pyroptosis, reduced myocardial injuries thus improved haemodynamics and cardiac function in AMI rats.	[119]
		Posttreatment with Ginsenoside Rh2 4 mg/kg/day IP for 2 weeks (compared with Sham)	↓ HR ↓ SBP, DBP, MAP ↓ LVESP, LVEDP	↑ NLRP3 ↑ Caspase 1 ↑ GSDMD	↑↑ IL‐1β	↑ LDH ↑ CK ↑ CK‐MB		
C57BL/6 J mice	Permanent LAD ligation 4 weeks	Treatment with Epigallocatechin gallate 50 mg/kg PO (duration not shown) (compared with Sham)	↑ %LVEF, %LVFS	↓ AIM2 ↓ ASC ↓ NLRP3 ↓ Cleaved caspase 1 ↓ GSDMD‐NT	↓ IL‐1β ↓ IL‐18	↓ %Fibrosis ↓ LDH ↓ CK‐MB	Epigallocatechin gallate mitigated cardiac pyroptosis by inhibiting LncRNA MEG3/TAF15/AIM2 axis thus improving cardiac function in MI rats.	[60]
SD rats	Permanent LAD ligation 4 weeks	Posttreatment with 2% H_2_ inhalant 3 h/day for 4 weeks after LAD ligation (compared with control)	↑ %LVEF, %LVFS ↓ LVIDd, LVIDs	↓ NLRP3 ↓ ASC ↓ Cleaved caspase 1 ↓ GSDMD‐NT	↓ IL‐1β	↓ %Fibrosis ↓ Cardiac tissue MDA ↓ ROS ↓ α‐SMA ↓ Fibronectin ↓ TGF‐β ↓ Collagen type I and III	H_2_ inhalant mitigated the expression of NLRP3‐mediated pyroptosis‐related protein, reduced the expression of fibrosis‐related proteins thus improved cardiac function and alleviates pathological remodelling in MI rats.	[79]
SD rats	Permanent LAD ligation 4 weeks	Posttreatment with MCC950 30 mg/kg/day IP for 4 weeks after LAD ligation (compared with control)	↑ %LVEF, %LVFS ↓ LVIDd, LVIDs	↓ NLRP3 ↓ ASC ↓ Cleaved caspase 1 ↓ GSDMD‐NT	↓ IL‐1β	↓ %Fibrosis ↔ Cardiac tissue MDA ↔ ROS ↓ α‐SMA ↓ Fibronectin ↓ TGF‐β ↓ Collagen type I and III	H_2_ inhalant mitigated the expression of NLRP3‐mediated pyroptosis‐related protein, reduced the expression of fibrosis‐related proteins thus improved cardiac function and alleviates pathological remodelling in MI rats.	[79]
C57BL/6 J mice	Permanent LAD ligation 4 weeks	Posttreatment with 2% H_2_ inhalant 3 h/day + MCC950 30 mg/kg/day IP for 4 weeks after LAD ligation (compared with control)	↑↑ %LVEF, %LVFS ↓ ↓ LVIDd, LVIDs	↓ NLRP3 ↓ ASC ↓ Cleaved caspase 1 ↓ GSDMD‐NT	↓ IL‐1β	↓↓ %Fibrosis ↓↓ Cardiac tissue MDA ↓↓ ROS ↓↓ α‐SMA ↓ Fibronectin ↓↓ TGF‐β ↓↓ Collagen type I and III	Astragaloside‐IV alleviated MI‐induced myocardial fibrosis and pathological cardiac remodelling by suppressing ROS/Caspase‐1/GSDMD signalling pathway in mice.	[91]
		Astragaloside‐IV 40 mg/kg/day PO for 4 weeks after LAD ligation (compared with control)	↑ %survival ↑ %LVEF	↓ NLRP3 ↓ Cleaved caspase 1 ↓ GSDMD‐NT	↓ IL‐1β ↓ Cleaved IL‐1β ↓ IL‐18 ↓ Cleaved IL‐18	↓ %Fibrosis ↓ Cardiomyocyte size ↓ ROS ↓ α‐SMA ↓ Collagen I		
C57BL/6 mice	Permanent LAD ligation 4 weeks	Posttreatment with GI‐Y1 50 mg/kg/day IG for 4 weeks after LAD ligation (compared with control)	↑↑ %LVEF, %LVFS	↓ GSDMD‐NT	↓ IL‐1β ↓ IL‐18	↓ Serum LDH ↓ Serum CK‐MB ↓ Collagen I ↓ TGF‐β	GI‐Y1 inhibited GSDMD‐mediated myocardial pyroptosis thus alleviated cardiac dysfunction and remodelling in MI mice.	[34]
SD rats	Permanent LAD ligation 8 weeks	Posttreatment with Tanshinone IIA 1.5 mg/kg/day PO for 8 weeks after LAD ligation (compared with control)	↑ %LVEF ↔ LVIDd ↓ LVIDs	↓ NLRP3 ↔ GSDMD ↓ GSDMD‐NT ↔ Caspase 1 ↓ Cleaved caspase 1	↓ IL‐1β ↓ IL‐18	↓ %Fibrosis ↓ Serum NT‐proBNP ↓ TUNEL positive nuclei ↓ TLR4 ↓ pNF‐κBp65	Tanshinone IIA inhibited myocardial pyroptosis through TLR4/NF‐κB p65 pathway thus alleviated cardiac dysfunction and remodelling in MI rats.	[117]
		Posttreatment with Captopril 10 mg/kg/day PO for 8 weeks after LAD ligation (compared with control)	↑ %LVEF ↓ LVIDd ↓ LVIDs	↓ NLRP3 ↔ GSDMD ↓ GSDMD‐NT ↔ Caspase 1 ↓ Cleaved caspase 1	↓ IL‐1β ↓ IL‐18	↓ %Fibrosis ↓ Serum NT‐proBNP ↓ TUNEL positive nuclei	Captopril inhibited myocardial pyroptosis through TLR4/NF‐κB p65 pathway thus alleviated cardiac dysfunction and remodelling in MI rats.	

Similar to in vitro studies, exosome treatments reported in animal models also showed a promising therapeutic avenue against IHD‐mediated pyroptosis. In mouse, rat and swine studies, exosomes administration have been shown to promote cardiac repair, reduce inflammation, mitigate adverse remodelling and enhance cardiac function [32, 37, 38]. Exosomes containing miR‐133a, miR‐320b, miR‐100‐5p, miR182‐5p and LncRNA GHET1 administration reduced canonical pyroptosis activation in cardiac I/R animals [32, 34, 35, 39, 40]. Furthermore, M2 exosome‐derived miR‐148a significantly attenuated TLR4/NF‐κB/NLRP3‐mediated pyroptosis, and alleviating myocardial pyroptosis, inflammation, fibrosis and cardiac dysfunction in cardiac I/R rats [36]. These findings highlight the therapeutic potential of exosome‐based interventions in targeting pyroptosis and associated pathological processes in IHD. Further research and clinical translation are warranted to optimise exosome‐based therapies in improving cardiovascular health.

## 9 Roles of Pyroptosis in Nonischaemic Heart Diseases

Previous studies have reported that ischaemic and nonischaemic heart diseases share common phenotypic characteristics in the development of myocardial dysfunction, adverse remodelling and heart failure in large mammalian models and humans [128, 129]. However, the precise mechanistic insights remain largely unexplored. In addition to the well‐established role of pyroptosis in IHD, recent studies increasingly demonstrate that GSDMD‐mediated pyroptosis also plays a crucial role in the development of heart failure in nonischaemic heart disease. Notably, pyroptosis has been identified in the pathogenesis of cardiotoxicity, cardiac hypertrophy, dilated cardiomyopathy, sepsis‐induced cardiomyopathy, arrhythmia and cardiac ageing [130, 131, 132, 133, 134, 135, 136, 137, 138]. Previous studies have reported that doxorubicin, an anthracycline chemotherapeutic agent, induced cardiotoxicity through the canonical and noncanonical pyroptosis, resulting in myocardial injury, contractile dysfunction and heart failure [134, 137, 139]. Additionally, doxorubicin has also been reported to directly bind to GSDMD, disrupting its autoinhibition, promoting lipid membrane oligomerisation and facilitating GSDMD‐mediated myocardial pyroptosis [137]. Furthermore, previous studies have demonstrated that pressure overload and angiotensin II stimulation could lead to abnormal activation of canonical pyroptosis, contributing to cardiac fibrosis, hypertrophic cardiomyopathy and the progression of heart failure [132, 140]. Specifically, angiotensin II stimulation and pressure overload induced by transverse aortic constriction (TAC) caused mitochondrial dysfunction and activation of the stimulator of interferon genes (STING) pathway, triggering inflammasome assembly and canonical pyroptosis [132]. In contrast, pharmacological and genetic ablation of caspase 1 and GSDMD could mitigate pressure overload‐induced cardiac hypertrophy [132, 140].

## 10 The Influent of Gender and Age on Myocardial Pyroptosis

The influence of gender and age on myocardial pyroptosis is a critical area of research in cardiovascular pathology. It is well established that, in age‐matched controlled studies, women are less susceptible to the incidence of IHDs compared to men, except in women aged 65 years or older [141, 142, 143, 144]. Limited studies have explored the role of oestrogen in attenuating pyroptosis across various organs. In the liver, oestrogen has been shown to protect against lipid and sepsis‐induced hepatic damage by modulating oxidative stress and caspase 1‐mediated pyroptosis [145, 146]. In the heart, Meng et al. [147] demonstrated that treatment with 17β‐oestradiol, a major female hormone, conferred cardiac protection through the activation of oestrogen receptor α‐mediated autophagy in the cardiac aortic endothelial cells of apolipoprotein E‐deficient (apoE^−/−^) mice. Additionally, they observed that bilateral ovariectomy, which induced oestradiol deprivation, exacerbated pyroptosis in the same model [147]. On the other hand, age plays a significant role in the progression of myocardial diseases, as older individuals tend to have an enhanced inflammatory state that may accelerate myocardial pyroptosis. Liu et al. [148] demonstrated that the activation of NLRP3 facilitated canonical pyroptosis in D‐galactose‐induced cardiac ageing. Interestingly, administration with NLRP3 inhibitor (MCC950) or caspase 1 inhibitor (VX‐765) could ameliorate pyroptosis activation in both oestrogen deprivation and cardiac ageing models [147, 148]. Understanding these factors is crucial for elucidating the role of pyroptosis in cardiac pathologies and for developing novel therapeutic strategies that can mitigate heart pathologies and improve clinical outcomes across various demographic groups.

## 11 Conclusions

The evidence across in vitro, in vivo and clinical research indicates that, upon the progression of IHD, there was extensive release of inflammatory mediators and DAMPs, which induced the priming of inflammasomes predominantly through TLR4/Myd88/NF‐κB/p65 signalling cascades. These result in the catalysis of the proteolytic cleavage of GSDMD‐NT‐mediated pyroptosis. Since adult cardiomyocytes are limited as regards their regenerative capacity when it comes to replacing the damaged myocardium, IHD‐mediated cardiomyocyte pyroptosis would lead to the loss of functional myocardium, adverse cardiac remodelling, contractile dysfunction and the development of postischaemic heart failure. Also, emerging evidence increasingly suggests a mechanistic crosstalk between mitochondrial abnormalities and pyroptosis in the context of IHD. Interventions aimed at targeting GSDMD‐mediated pyroptosis signalling cascades have demonstrated promising therapeutic benefits against IHD‐related pathologies in preclinical settings. The specific mechanisms underlying pyroptosis, as well as the identification and the broader involvement of mitochondria and crosstalk with other forms of programmed cell death in IHD are current areas of ongoing research. Further studies are necessary to allow us to completely understand the role of pyroptosis in IHD and its potential implications for future therapeutic interventions.

## Author Contributions

**Chanon Piamsiri:** data curation (lead), formal analysis (lead), investigation (lead), methodology (equal), validation (lead), visualization (lead), writing – original draft (lead). **Chayodom Maneechote:** data curation (supporting), formal analysis (supporting), methodology (equal), supervision (supporting), validation (supporting), visualization (supporting), writing – review and editing (supporting). **Siriporn C. Chattipakorn:** conceptualization (equal), funding acquisition (equal), investigation (supporting), methodology (supporting), project administration (supporting), supervision (supporting), validation (supporting), visualization (supporting), writing – review and editing (supporting). **Nipon Chattipakorn:** conceptualization (lead), funding acquisition (lead), investigation (equal), methodology (equal), project administration (lead), resources (supporting), supervision (lead), validation (lead), visualization (lead), writing – review and editing (lead).

## Conflicts of Interest

The authors declare no conflicts of interest.

## Data Availability

Data are available upon reasonable request from the corresponding author.
